# The origins of mammal growth patterns during the Jurassic mammalian radiation

**DOI:** 10.1126/sciadv.ado4555

**Published:** 2024-08-07

**Authors:** Elis Newham, Ian J. Corfe, Philippa Brewer, Jen A. Bright, Vincent Fernandez, Neil J. Gostling, Simone Hoffmann, Kai R. K. Jäger, Erika Kague, Goran Lovric, Federica Marone, Elsa Panciroli, Philipp Schneider, Julia A. Schultz, Heikki Suhonen, Alex Witchell, Pamela G. Gill, Thomas Martin

**Affiliations:** ^1^ School of Engineering and Materials Sciences, Queen Mary University of London, London, UK.; ^2^ Section Palaeontology, Bonn Institute of Organismic Biology, Rheinische Friedrich-Wilhelms-Universität Bonn, Bonn, Germany.; ^3^ Institute of Biotechnology, University of Helsinki, Helsinki, Finland.; ^4^ Research Laboratory, Geological Survey of Finland, Espoo, Finland.; ^5^ Department of Science, Natural History Museum, London, UK.; ^6^ School of Natural Sciences, University of Hull, Hull, UK.; ^7^ European Synchrotron Radiation Facility, Grenoble, France.; ^8^ School of Biological Sciences, Faculty of Environmental and Life Sciences, The University of Southampton, Southampton, UK.; ^9^ Department of Anatomy, College of Osteopathic Medicine, New York Institute of Technology, Old Westbury, NY, USA.; ^10^ School of Physiology, Pharmacology and Neuroscience, Biomedical Sciences, University of Bristol, Bristol, UK.; ^11^ Centre for Genomic and Experimental Medicine, Institute of Genetics and Cancer, The University of Edinburgh, Edinburgh, UK.; ^12^ Swiss Light Source, Paul Scherrer Institute, Villigen, Switzerland.; ^13^ National Museums Scotland, Chambers Street, Edinburgh, UK.; ^14^ Oxford University Museum of Natural History, Parks Road, Oxford, UK.; ^15^ Bioengineering Science Research Group, Faculty of Engineering and Physical Sciences, University of Southampton, Southampton, UK.; ^16^ High-Performance Vision Systems, Center for Vision, Automation & Control, AIT Austrian Institute of Technology, Vienna, Austria.; ^17^ Department of Physics, University of Helsinki, Helsinki, Finland.; ^18^ School of Earth Sciences, University of Bristol, Bristol, UK.

## Abstract

We use synchrotron x-ray tomography of annual growth increments in the dental cementum of mammaliaforms (stem and crown fossil mammals) from three faunas across the Jurassic to map the origin of patterns of mammalian growth patterns, which are intrinsically related to mammalian endothermy. Although all fossils studied exhibited slower growth rates, longer life spans, and delayed sexual maturity relative to comparably sized extant mammals, the earliest crown mammals developed significantly faster growth rates in early life that reduced at sexual maturity, compared to stem mammaliaforms. Estimation of basal metabolic rates (BMRs) suggests that some fossil crown mammals had BMRs approaching the lowest rates of extant mammals. We suggest that mammalian growth patterns first evolved during their mid-Jurassic adaptive radiation, although growth remained slower than in extant mammals.

## Teaser

X-ray imaging of dental growth rings reveals life history differences between living mammals and their Jurassic ancestors.

## INTRODUCTION

**Fig. 1.**
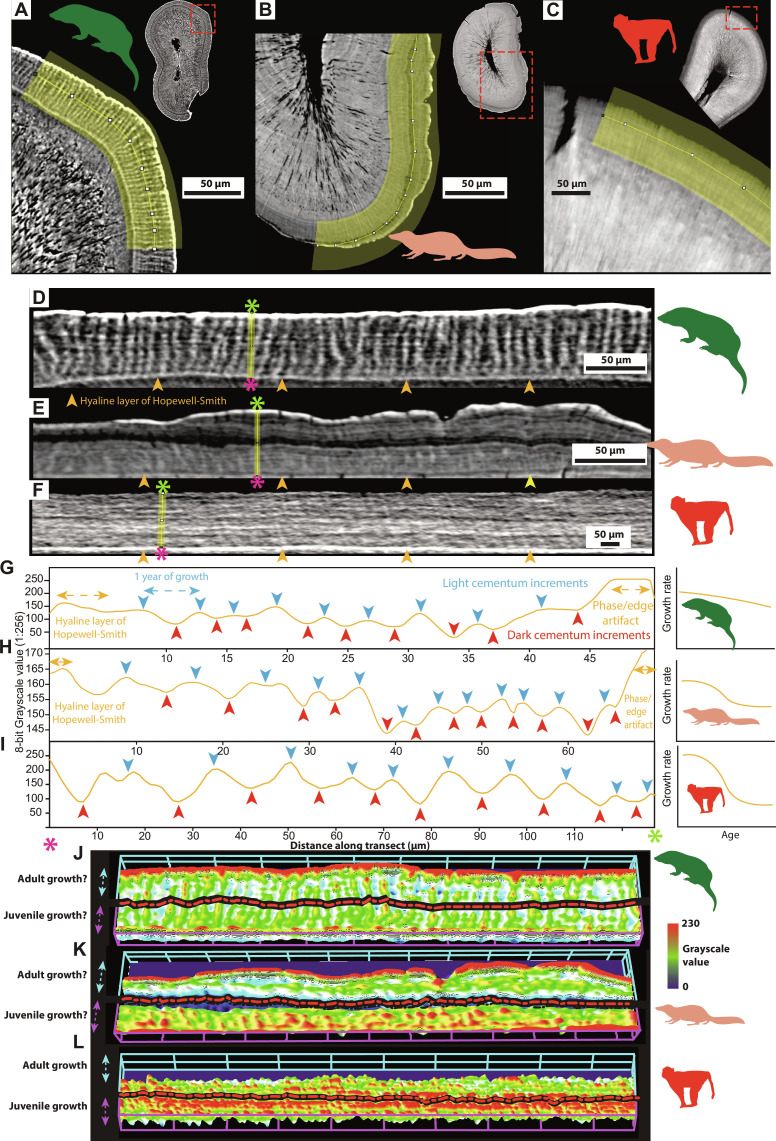
Comparison between cementum growth in nonmammalian mammaliaforms, early mammals, and extant mammals. (**A** to **C**) Details of the cementum (highlighted) of (A) docodontan *Krusatodon* NHMUK PV M36541, (B) cladotherian *Dryolestes* Gui Mam 1191, and (C) primate *M. mulatta* k39. (**D** to **F**) Straightened sections from (A) to (C), respectively, including the external-most dentine hyaline layer of Hopewell-Smith (light phase indicated by orange arrows) used for measuring cementum increment widths along eight evenly spaced radial transects (yellow bars for examples) between the inner cementum boundary/cementum-dentine junction (pink asterisks) and outer cementum boundary/root surface (green asterisks). (**G** to **I**) Mean grayscale values (SRCT data) measured across 10-pixel-thick transects in (D) to (F), respectively; light increments forming peaks, and dark increments forming troughs. Annual growth measured as the spacing between light increments (Materials and Methods) (fig. S1). Schematic patterns for spacing/growth rate through life represented in the right-hand summary charts. (**J** to **L**) 3D surface plots of grayscale texture in (D) to (F), with differences between known [for (L)] and estimated [for (K)] juvenile versus adult cementum texture highlighted using black dashed-dotted lines (see fig. S1). Black dashed-dotted line in (J) represents periods of life subsampled to test for differences in juvenile versus adult cementum in mammaliaforms. All silhouettes obtained from PhyloPic (http://phylopic.org/) under a public domain license.

**Fig. 2.**
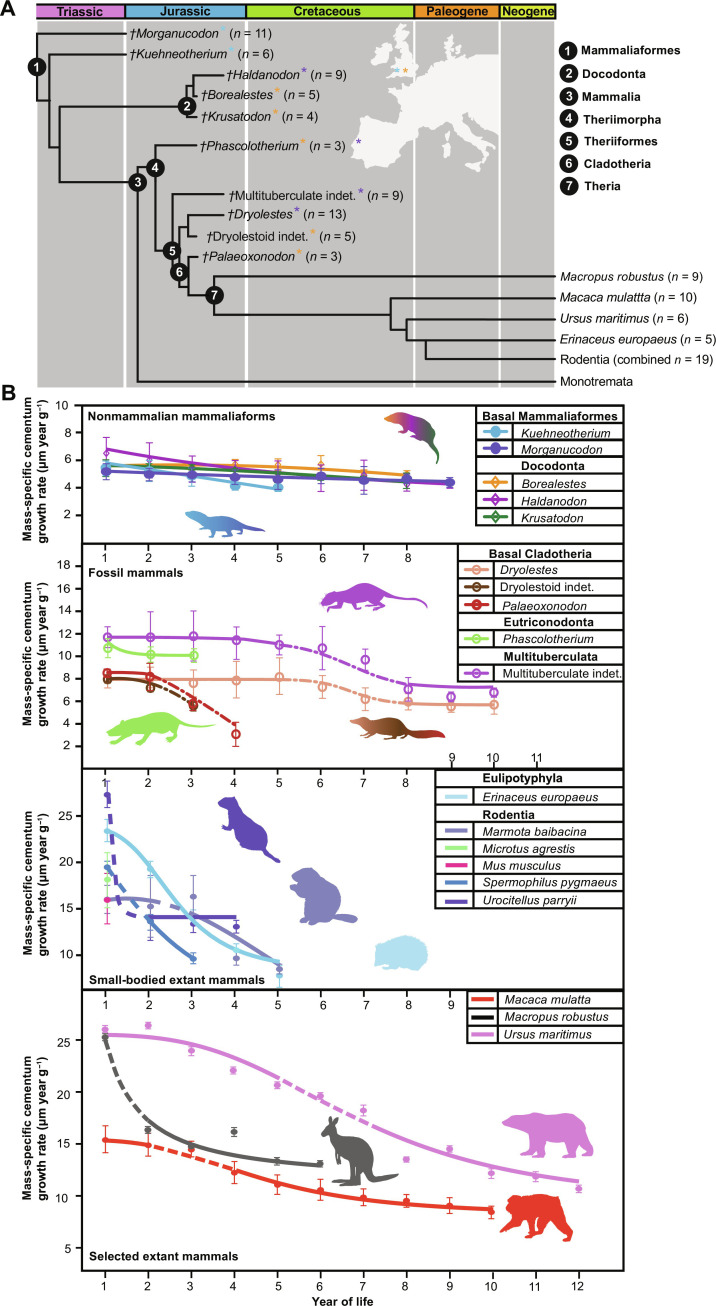
msGRs through life measured for fossil mammaliaforms and selected extant mammals. (**A**) Time-calibrated phylogeny with major groups, sample number, and origin highlighted. (**B**) msGR curves for nonmammalian mammaliaforms, fossil mammals, and selected extant mammals. Data restricted to years of life represented by ≥3 specimens (for full life-span estimates, see Fig. 3). Dashed portions of extant plots represent the mean period for attainment of sexual maturity for the respective taxon. Bracketed vertical lines represent SDs for subsampled measurements (Materials and Methods). Symbols (see legends) represent mean msGRs for the respective taxon during the respective year of life. Lines for all taxa represent best-fitting nonlinear models (Table 1). Dashed-dotted portions of fossil crown mammal plots represent estimated periods for attainment of sexual maturity. Extant mammals included were chosen due to their comparable sampled life spans and body masses to fossil mammaliaforms and phylogenetic diversity; see fig. S5 for full extant sample. Source data are provided as data S1. Time-calibrated Mesozoic mammaliaform phylogeny sourced from Araújo *et al.* (*8*). Extant mammal phylogeny sourced from Vertlife (http://vertlife.org/). All silhouettes obtained from PhyloPic (http://phylopic.org/) under a public domain license.

The physiological maintenance of consistent body temperatures above ambient levels in mammals results in basal metabolic rates (BMRs) higher than equivalent resting metabolic rates in ectothermic reptiles and amphibians (*1*–*9*). High BMRs allow for rapid skeletal growth in juveniles that slows with the attainment of sexual maturity [which generally occurs significantly earlier than in ectothermic vertebrates (*10*–*15*)]. This creates sigmoidal growth rate patterns through life (Figs. 1 and 2) in each of the three major extant mammalian clades: Placentalia (*16*), Marsupialia (*2*, *16*), and Monotremata (*13*). Often classed as “determinate” growth patterns, the mammal (and avian) condition broadly differs from determinate growth patterns in other extant vertebrates (e.g., lepidosaurs) due to the steeper truncation from exceptionally rapid juvenile growth rates to minimal adult growth over a relatively short period of time. The BMR remains elevated in sexually mature mammals, which is believed to limit the maximum life span of mammals compared to ectotherms of similar size and ecology due to increased rates of metabolic oxidative stress (*6*, *9*). Understanding how this characteristic suite of life history parameters—life span, growth rates, and growth patterns—developed through the fossil record is critical for our understanding of mammalian physiological evolution.

Numerous studies have sought to identify growth patterns in fossil mammals and their synapsid relatives (*17*–*21*), concluding that mammaliaforms (stem lineage mammals) may have retained labile growth patterns and slow juvenile growth rates (relative to extant mammals) through the Mesozoic (*18*). Conflicting conclusions, from mandibular size and links with the origin of diphyodonty (a single replacement of teeth, autapomorphic to Mammaliaformes; Materials and Methods) (*4*, *19*, *21*), have been used to suggest that mammalian determinate growth (earlier and steeper truncation in growth rates) evolved on the mammalian stem lineage. However, these interpretations have lacked the crucial aspect of a time frame, i.e., life span. Without evidence of the individual age of the specimens studied, the reported growth changes cannot be calibrated over the life span of the respective taxa, and it is not feasible to estimate the true growth rates. This undermines the ability to accurately distinguish growth patterns and compare them to those of extant mammals.

This limit was recently overcome through the application of cementochronology, using synchrotron radiation–based x-ray computed tomography (SRCT) (*4*). Dental cementum, the mineralized tissue surrounding tooth roots and connecting them to the periodontal ligament, is unique among mammalian hard tissues as its growth is continuous throughout life and it is not remodeled and rarely resorbed, and annual increments can be counted (*22*, *23*) (Fig. 1). Here, we demonstrate how counting cementum growth layer groups (Figs. 1 and 2) and analyzing their radial thickness and texture (fig. S1) can serve as a surrogate measure for mammal life span, growth rate, and growth pattern. A critical time in the evolution of these life history parameters was across the Jurassic mammalian adaptive radiation (*24*, *25*), and so we examine fossilized cementum increments in stem and crown lineage mammals from three localities, each within either the Early, Middle, or Late Jurassic. This has provided an unparalleled opportunity to study samples that span the mid-Jurassic radiation both phylogenetically and temporally, in quantities sufficient for the population-level sampling necessary to characterize life history.

### Cementochronology of Mesozoic mammaliaforms

Cementochronology is an important tool to investigate life history (*26*–*32*). Nondestructive SRCT imaging allows counting of annual growth layer groups [a pair of one thick “light,” higher density increment deposited during favorable seasons and one thin “dark,” lower density increment deposited during unfavorable seasons (*26*, *27*)] deposited for each year of life in the acellular extrinsic fiber portion of the cementum tissue (AEFC) (Materials and Methods) (Fig. 1). Validation studies performed on a wide range of extant (*22*, *26*) mammals of known age suggest that this tissue comprises increments with strong circum-annual periodicity (*23*), and herein is the tissue referenced when using the term “cementum” (see Materials and Methods for validation of annual periodicity using dentary bone lines of arrested growth in fossil mammaliaforms).

The first application of cementochronology to study the physiological evolution of Mesozoic mammals used SRCT in population-sized samples of the Early Jurassic nonmammalian mammaliaforms *Morganucodon* and *Kuehneotherium* (*4*). This revealed unexpectedly long life spans for their body mass and reptile-like physiology. Both life span and BMR covary with body mass among extant mammals, with larger taxa having lower mass-specific BMRs (msBMRs) and longer life spans (*4*–*6*, *9*) [although this relationship is complicated among flying/gliding and marine taxa due to their specific ecological requirements; see correspondence between Meiri and Levin (*5*) and Newham *et al.* (*4*, *6*)].

Here, we demonstrate how not only counts for life span (Figs. 1 and 2) but also further analysis of the radial thickness and texture (fig. S1) of cementum growth layer groups can serve as a surrogate measure for growth rate and growth pattern. Cementum helps to keep tooth crowns level above the gumline through life as the jaw remodels through growth and crowns are worn from occlusion (*22*, *26*). This is reflected in frequent observations of changes in increment width and quality at the reduction of somatic growth and attainment of sexual maturity in extant mammals (*22*, *27*–*32*). A number of extant mammal species display a negative sigmoidal trend in cementum increment thickness that follows their somatic growth patterns: rapid juvenile growth is represented by widely spaced irregularly organized increments (*28*), and the advent of sexual maturity and cessation of somatic growth lead to narrowly spaced, more uniformly organized increments (Figs. 1 and 2) (*22*, *27*–*31*).

To study the evolution of these life history parameters across the Jurassic mammalian adaptive radiation (*24*, *25*), we analyze the cementum of diphyodont fossil mammaliaforms from three localities that originate between the Early-to-Late Jurassic. The Early Jurassic (Hettangian) *Hirmeriella* fissure suite (Wales, United Kingdom) includes some of the earliest known nonmammalian mammaliaforms (*33*); the Middle Jurassic Forest Marble fauna (Bathonian) of Oxfordshire (United Kingdom) includes one of the most taxonomically diverse known faunas of cohabiting nonmammalian mammaliaforms (docodontans) and early theriimorph mammals (*34*) (therians and all extinct taxa more closely related to therians than to monotremes); and the Kimmeridgian Guimarota fauna (Portugal) provides the most numerous Late Jurassic fossils of nonmammalian mammaliaforms and theriiform mammals currently known (*35*) (Fig. 2; see Materials and Methods).

We present a method for quantifying growth rates of cementum growth layer groups in volumetric SRCT datasets by counting and measuring distances (in micrometers) between peaks in grayscale values (representative of light circum-annual cementum increments) along radial transects through the cementum in preprocessed and subsampled SRCT slices (Fig. 1; Materials and Methods) (*27*, *36*). Counts are used to estimate life spans (years) (fig. S1) and msBMRs (ml O_2_ hour^−1^ g^−1^) following the method outlined by Newham *et al.* (*4*) (fig. S2). To allow comparison of growth rates between fossil and extant clades of varying body mass, mean width measurements for each year of life were processed to create mass-specific annual growth rates (msGRs; μm year g^−1^). This was performed using a mass-specific growth ratio created by exponential phylogenetically informed least-squares (PGLS) regression (*37*) between log-transformed body mass (in grams) and log-transformed width of the first cementum growth layer group (in micrometers) in a sample of 23 extant therian mammals (fig. S3).

## RESULTS

### Growth patterns of Jurassic mammaliaforms

All studied nonmammalian mammaliaforms show slow initial/juvenile msGRs and less pronounced changes in growth patterns through life compared to theriimorph mammals (Figs. 1 and 2, and Table 1). Jurassic theriimorph mammals have elevated early growth rates, typical of mammalian growth, but initial msGRs are still lower than those of extant therians (marsupials and placentals) (Fig. 2, Table 1, and fig. S5). Significant differences were found between the pooled msGRs of nonmammalian mammaliaforms and fossil theriimorph mammals for every year of life when compared using analysis of variance (ANOVA) (*F* = 91.85, *P* < 0.001). While both groups showed a decrease in increment width through life, nonmammalian mammaliaforms showed significantly slower growth rates through their first 6 years compared to fossil crown mammals (Fig. 2) (ANOVA *F* = 75.12, *P* < 0.001). The low maximum slope and starting values for nonlinear models fitted for each nonmammalian mammaliaform taxon (Fig. 2 and Table 1) suggest a relatively slow and consistent decrease in growth through life. In contrast, fossil theriimorph mammals provide elevated initial growth rates followed by relatively steep declines later in life (Fig. 2 and Table 1). This pattern is typified by the sigmoidal models fitted to the data for the Late Jurassic *Dryolestes* and the pooled Late Jurassic multituberculates, with elevated msGRs until ages of 5 and 6 years and fastest declining rates until years 8 and 9, respectively (Fig. 2). We infer that the nonsigmoidal growth patterns of *Palaeoxonodon* and the pooled Middle Jurassic dryolestidans could be caused by a lack of fully adult specimens available in our sample (Supplementary Note 1) and that the full life-span range for *Palaeoxonodon* would show a sigmoidal growth pattern.

**Table 1.** Cementum growth and life history variables for fossil mammaliaforms and selected extant mammals. “Max. sample life span” refers to the maximum life span known for the studied subsample of specimens in preservational categories a and b, as opposed to the maximum known wild life span of the respective taxon (“max. known wild life span”). See table S1 for full extant sample. All fossil data and data for *M. mulatta* and *E. europaeus* are from SRCT. All other data are from histology. See table S1 for all extant mammals studied and growth model function and statistics. Na, not applicable.

Taxon (*n*)	Max. sample life span (years)	Max. known wild life span (years)	Mean known or estimated body mass (g)	Sexual maturity min./max. age (years)	Cementum growth rate reduction min./max. age (years)	First/final year msGR (μm year g^−1^)	Best-fitting cementum growth model
*Morganucodon* (*11*)	14	Na	17.9	Na	Na	5.22/4.42	Quadratic
*Kuehneotherium* (*6*)	9	Na	23.8	Na	Na	5.45/4.05	Quadratic
*Krusatodon* (*4*)	8	Na	45.8	Na	Na	5.43/4.51	Gaussian
*Borealestes* (*5*)	9	Na	32.2	Na	Na	5.40/5.03	Gaussian
*Haldanodon* (*9*)	12	Na	64	Na	Na	6.47/4.26	Exponential
*Dryolestes* (*13*)	11	Na	130	Na	5/7	7.98/5.68	Hill’s sigmoidal
Dryolestoid indet. (*5*)	4*	Na	25	Na	3/4	7.94/5.61	Quadratic
*Palaeoxonodon* (*3*)	5	Na	16.9	Na	Na	8.35/4.22	von Bertalanffy
*Phascolotherium* (*3*)	3*	Na	70.8	Na	2/3	10.8/9.94	Exponential
Multituberculate indet. (*9*)	11	Na	45	Na	5/7	11.67/8.41	Hill’s sigmoidal
*E. europaeus* (*5*)	8	8	750	0.9/1	1/3	23.6/7.99	Hill’s sigmoidal
*M. mulatta* (*10*)	10	36	8240	2/4	1/4	15.0/8.85	Hill’s sigmoidal
*Ursus maritimus* (*6*)	15	22	300,000	3/7	3/7	26.3/10.8	Hill’s sigmoidal
*Macropus robustus* (*9*)	8	22	30,000	1/2	1/2	25.7/11.2	Hill’s sigmoidal
*Marmota baibacina* (*3*)	5	14	5250	2/3	2/4	16.216/10.119	Hill’s sigmoidal
*M. agrestis* (*4*)	1	1	30	<1/<1	Na	19.026/19.026	Na
*M. musculus* (*5*)	1	1	20.5	<1/<1	Na	16.020/16.020	Na
*Spermophilus pygmaeus* (*4*)	3	7.1	250	1/2	1/2	19.705/9.845	Quadratic
*Urocitellus parryii* (*3*)	5	10	1500	1/2	1/2	27.545/16.588	Hill’s sigmoidal

*Maximum age estimate found but not the maximum life-span estimate for the respective fossil taxon.

Comparison between fossil and extant data places evolutionary change between Jurassic taxa into a more complete physiological perspective (Fig. 2 for extant taxa with comparable sampled life spans to fossil mammaliaforms from diverse mammalian orders; fig. S5 for full sample). While extant therians show a substantially higher diversity in growth patterns than our fossil sample, all have initial msGRs that exceed those of any Jurassic mammal and have significantly higher pooled values than their fossil theriimorph relatives (ANOVA *F* = 44.55, *P* < 0.001). Most extant species show sigmoidal growth, with considerably steeper growth rate reductions than *Dryolestes* and substantial differences between growth in early versus later life (Fig. 2, fig. S5, and table S1).

### Life spans and BMRs of Jurassic mammaliaforms

**Fig. 3.**
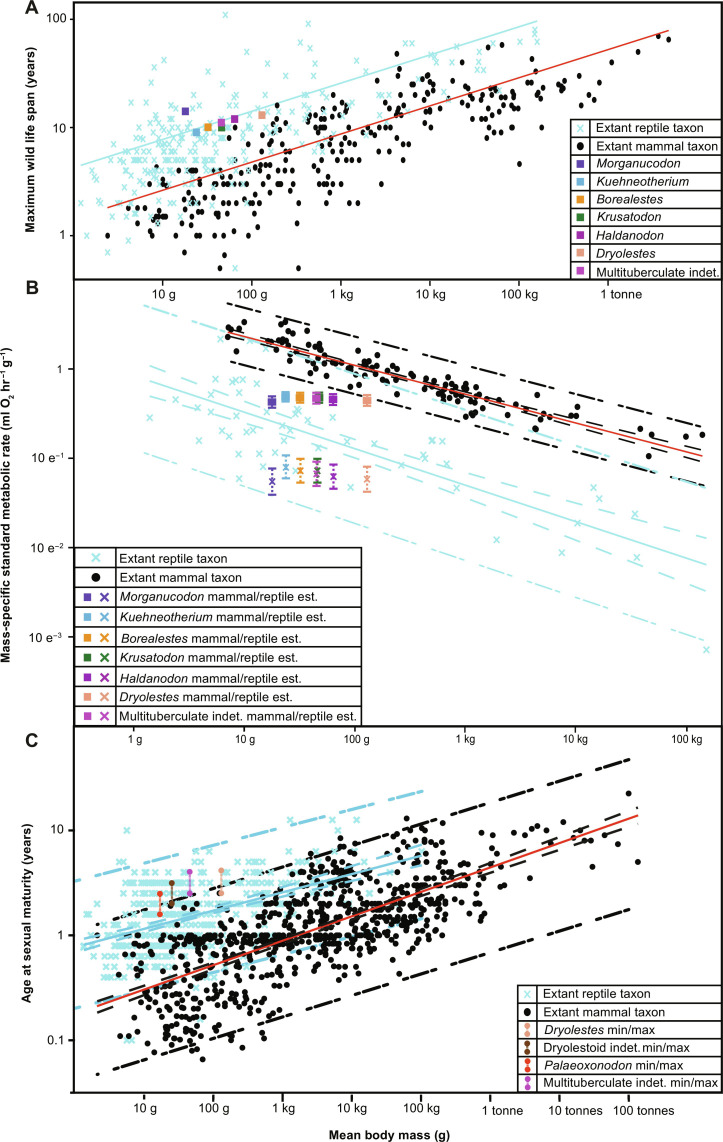
Life span, metabolic rate, and age at sexual maturity estimates for fossil mammaliaforms. (**A**) Log_10_ PGLS biplot of mean body mass (g) against maximum wild life span (years) for extant mammals (*n* = 279 taxa), non-avian reptiles (*n* = 252 taxa) [data, Newham *et al.* (*4*)], and fossil mammaliaforms. (**B**) Log_10_ PGLS biplot of mean body mass (g) against msSMR (ml O_2_ hour^−1^ g^−1^) for extant mammals (*n* = 117 taxa), non-avian reptiles (*n* = 55 taxa) [data, Newham *et al.* (*6*)], and respective estimates for fossil mammaliaforms using life span/SMR regression for both extant clades (extended data). Square fossil values estimated using the extant mammal regression; “X” fossil values estimated using the extant reptile regression. (**C**) Log_10_ PGLS biplot of mean body mass (g) against mean age at sexual maturity (years) for extant mammals (*n* = 777 taxa), non-avian reptiles (*n* = 411 taxa), and estimates for fossil mammals. PGLS regression lines [(A) to (C)] for extant mammals (red) and extant reptiles (blue), 95% CIs [(B) and (C)] represented by dashed lines, and 95% predictor intervals represented by dotted lines. Data for extant taxa, *Kuehneotherium*, and *Morganucodon*, Newham *et al.* (*6*). Source data provided as data S1.

Maximum life-span estimates for all fossil species are longer than those of extant terrestrial taxa of comparable size (table S1 and Fig. 3A; crown mammals are subsampled to include only clades with life spans extending beyond the point of fastest growth rate reduction). Estimation of BMRs using these maximum life spans (fig. S3) and PGLS (*4*) comparison with extant mammals and non-avian reptiles suggest that nonmammalian mammaliaforms and multituberculates retained BMR levels lower than could be predicted for any extant wild terrestrial mammal of comparable size (Fig. 3, A and B). The fossil cladotherian *Dryolestes* occupied an intermediate grade closest to (and potentially overlapping when including its confidence limits) the lowest predicted range for extant mammals although outside the mammalian measured range. This supports predictions of increasing BMR levels from nonmammalian mammaliaforms to fossil cladotherian fossil mammals and extant mammals (*1*, *4*).

### Attainment of sexual maturity

**Fig. 4.**
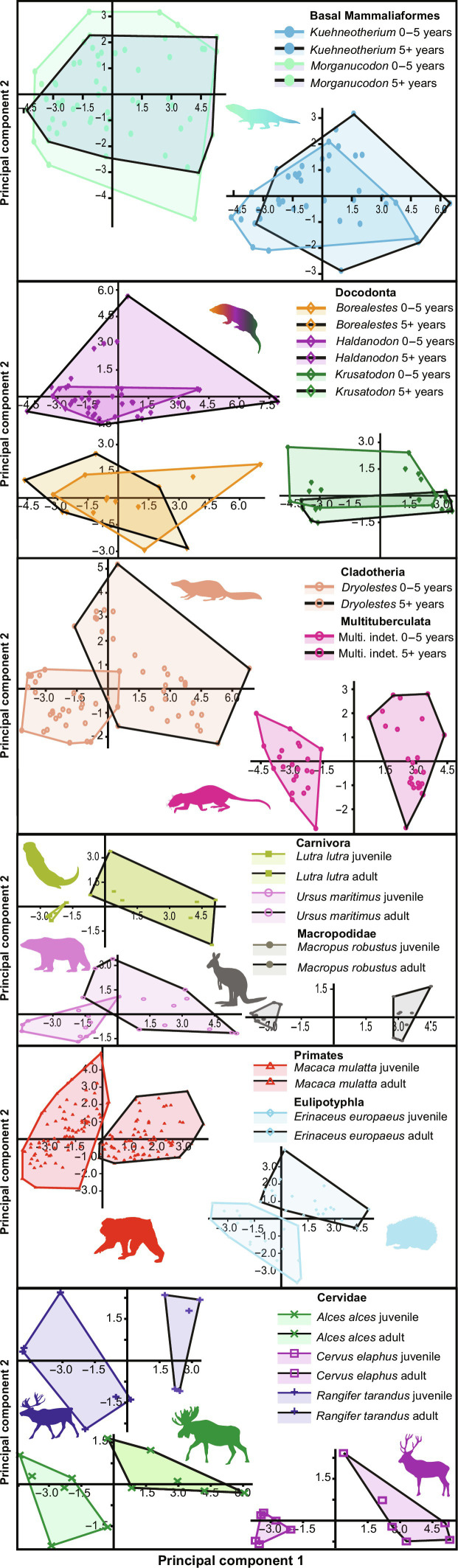
Statistical comparisons between juvenile versus adult cementum texture for fossil mammaliaforms and extant mammals. PCA scores for 14 3D surface profiling measures applied to data of juvenile and adult cementum for extant mammals and cementum deposited before/after 5 years of life for fossil mammals (the mean point of growth rate truncation found for fossil crown mammals). Juvenile data bound by colored-rimmed convex hulls, and adult data bound by black-rimmed convex hulls. Principal component 1 represents grayscale contrast and spatial distribution, with positive values reflecting greater contrast and increased anisotropy. Principal component 2 represents “functional” (surface roughness) factors, with positive values showing greater grayscale variation within the SD of the sample (fig. S1). See table S3 and Supplementary Note 3 for further description of profiling measures and analysis. Source data are provided as data S1. All silhouettes obtained from PhyloPic (http://phylopic.org/) under a public domain license.

Each extant mammal species studied displayed a period of inflected msGRs (increased slope between consecutive years of life; Table 1) that bound the known range for its age at sexual maturity (Fig. 2, table S1, and fig. S5). This posed the question of whether the inflection in growth found for fossil theriiform mammals (Late Jurassic multituberculates and *Dryolestes*) (Fig. 2) also correlates with the attainment of sexual maturity (*22*, *27*, *28*, *30*). To test this, we quantitatively compared the structure and texture of juvenile and adult cementum in eight extant taxa using three-dimensional (3D) surface profiling techniques (Materials and Methods). Principal components analysis (PCA) of this data for each taxon reveals that, in extant therians, juvenile and adult cementum occupy distinct regions of “texture space,” with adult data showing higher grayscale contrast, greater anisotropy, and less grayscale noise than juvenile data (Figs. 1, I and J, and 4 and fig. S1). This represents the reduction in growth rates at the attainment of sexual maturity, creating increments that are narrower (higher counts of contrasting light/dark increments deposited per unit area) and vary less in orientation and opacity (intraspecific variation in growth rates between each growth increment), providing quantitative data for the relationship between the attainment of sexual maturity and cementum growth previously only suggested qualitatively (Fig. 1) (*22*, *28*, *29*, *32*).

The application of the same texture measures to cementum data sampled before and after the inflection point of cementum growth rates in Late Jurassic theriiform mammals (*Dryolestes* and multituberculates) also creates distinct regions of texture space occupation when compared using PCA. However, for *Morganucodon*, *Kuehneotherium*, and docodontans, there is considerable overlap and no discrete texture space occupation (Fig. 4). These differences in between nonmammalian mammaliaforms and fossil theriiform mammals suggest that the truncation of growth in theriiforms corresponds to the attainment of sexual maturity in extant mammals (Fig. 2). Resultant estimates for age at sexual maturity (generated as a range between the earliest and latest age at sexual maturity relative to age at growth rate truncation found for our living mammal sample; table S1) were compared to data for extant mammals (*15*) (*n* = 774) and non-avian reptiles (*38*) (*n* = 411) by regressing against estimated body mass using PGLS (Fig. 3C). Phylogenetic analysis of covariance (ANCOVA) comparisons did not find different regression slopes for extant mammals and reptiles (*P* = 0.32), while the mean age at sexual maturity is significantly higher for reptiles (*P* < 0.05). Multituberculates are within the extant reptile range of age at sexual maturity relative to body size, and their youngest estimate aligns with the upper extant mammal predictor interval, whereas fossil cladotherians span extant reptile and extant mammal predictor and measured ranges (Fig. 3C). This mirrors the lower BMR estimates for the multituberculates versus the more derived *Dryolestes* (Fig. 3B) and conforms to the relatively slow pace of life history in early theriimorph mammals suggested by their growth rates, patterns, and life spans. As nonmammalian mammaliaforms did not produce the same sigmoidal growth patterns and showed no significant differences in cementum texture between increments formed before and after the same age of growth rate truncation as our Jurassic theriiform mammals, their age at sexual maturity could not be estimated here.

## DISCUSSION

### Jurassic mammaliaforms on the physiological spectrum

Our results add crucial detail to the evolution of life history across the Jurassic mammalian adaptive radiation. Although our cementum analysis methods are currently limited in use to diphyodont mammaliaform taxa and so conclusions bound to evolution within Mammaliaformes, an evolutionary transition of physiology to a more “mammalian” life history among Middle Jurassic theriiform mammals (fig. S6) supports a delayed acquisition of fully modern mammalian physiological traits suggested by other recent studies (*1*, *4*, *8*, *11*, *39*). Many traits related to endothermy appeared before the mid-Jurassic, but only consistently reached values approaching or within the range of extant mammals during this time. For example, it has been evidenced from long bone histology that longevity and age at sexual maturity both decreased in nonmammaliaform therapsid clades that survived the end-Permian mass extinction (*40*, *41*), with maximum estimated longevity (and so also inferred age at sexual maturity) falling to within 2 years in the earliest Triassic species of *Lystrosaurus* (*41*). Botha-Brink *et al.* (*41*) showed that both traits returned to values more comparable with those of pre-extinction taxa in clades radiating further into the Triassic. Also, while recent proxy data suggest that body temperatures in several lineages of nonmammalian mammaliaforms may have approached endothermic levels (*8*), they did not fall consistently within the extant mammalian range until the radiation of crown mammals. Although body temperature evolution has remained uncoupled from BMR evolution across the mammalian phylogeny (*7*), this is consistent with our predicted BMRs and evolutionary patterns in encephalization quotients (relative brain size) (*42*).

Determinate growth patterns themselves are not exclusively related to endothermy (*3*, *43*). However, the pattern of determinate growth combined with elevated juvenile growth rates shown here for extant mammal cementum and elsewhere for skeletal (*44*) and bodily growth (*10*) is indicative of their elevated, endothermic BMR values. This supports the hypothesis that Jurassic mammals had not developed life histories similar to extant terrestrial mammals of comparable body mass; comparatively rapid juvenile growth rates (fig. S5), sexual and skeletal maturity before 2 years of age (Fig. 3C), and maximum wild life spans of 7 years or less [aside from long-lived and secondarily dwarfed *Microcebus* primates (*45*)] (Fig. 3A).

Regarding nonmammalian mammaliaforms, we suggest that the apparent contradictions between our results and earlier interpretations of their growth patterns relate to a lack of absolute time frames available for studying morphological and histological data in previous studies (*18*, *19*). As qualitatively suggested for *Morganucodon* by O’Meara and Asher (*3*), we find that growth in later years of life is statistically slower than the first 2 years [the period estimated for the replacement of deciduous teeth (*4*)] of life for nonmammalian mammaliaforms studied here (ANOVA *F* = 11.89, *P* = 0.001), and the total reduction in growth rate through life is similar to those of several fossil theriimorph mammals. However, our ability to assign a year of life to each data point has allowed the pace and pattern of this growth rate reduction to be estimated across life history. We find that the reduction is spread over a considerable proportion of the entire life span of nonmammalian mammaliaforms with lower year-to-year reductions than in studied fossil crown mammals. Given the diversity of previous evidence for mammal-like growth in nonmammalian mammaliaforms, we do not suggest that they grew indeterminately but that they lacked the extant mammalian pattern of rapid juvenile growth rates [instead potentially following a pattern more akin to determinate growth in extant squamate reptiles (*3*, *43*)].

Recent findings of long gestation periods, precocial young, and early sexual maturity in the Early Paleocene placental mammal *Pantolambda* (*46*) and suggestions of similar life histories in Late Cretaceous and Early Paleocene multituberculates (*2*) have been used to suggest that life history strategies similar to placental mammals had been established by this period. Late Jurassic Guimarota multituberculates show the highest msGR values of our fossil sample, overlapping the extant mammal range and steeper decline at the attainment of sexual maturity than fossil cladotherian mammals (Fig. 2). This is consistent with qualitative bone histological results suggesting higher growth rates than coeval Cretaceous eutherian mammals (*18*) used to explain the evolutionary success of multituberculates (*47*). Our msGR results suggest that this mosaic of primitive (long life spans and intermediate BMRs) and derived (high juvenile msGRs) life history characters is part of a phylogenetically consistent increase in plasticity and variation in growth patterns and life spans among mammaliaforms and crown mammals (Supplementary Note 1) (*48*–*50*). This ranges from conserved patterns among lineages of nonmammalian mammaliaforms to contrasting patterns between early crown clades (conserved within clades as suggested by multituberculates and fossil cladotherians) and highly diverse patterns within and between living therian clades. This may be interpreted as a decoupling between life span and growth rate as an evolutionary innovation toward more labile life history strategies as part of the Jurassic radiation of crown mammals (*7*) (and subsequently expanded by later therians). Findings of physiologically intermediate life histories in clades nested phylogenetically between monotremes and extant therians raise questions regarding the potentially independent evolution of monotreme growth patterns and endothermic (although considerably more labile than most other mammals) physiologies (*13*).

Lower growth rates in the Guimarota taxa *Dryolestes* and the docodontan *Haldanodon*, relative to less derived crown mammals and nonmammalian mammaliaforms, respectively, and a delayed growth rate truncation point in *Dryolestes* relative to Forest Marble cladotherians may also suggest plasticity in these life history elements due to differences in environmental and ecological pressures between the Guimarota (*35*) and Forest Marble (*34*) biomes (Supplementary Note 1). Several examples are known of extant and extinct mammals and nonmammaliaform therapsids (see above), deploying flexible growth strategies in response to stressed or resource-limited environments (*40*, *48*, *49*), including substantial changes in the age of attainment of sexual maturity and completion of somatic growth (*14*, *49*, *50*). While this apparent inherent flexibility in physiological proxies may be potentially seen as a caveat of our study, we believe that the phylogenetic, temporal, and geographic range in our sampled taxa showing elevated longevity and age at sexual maturity compared to extant small-bodied mammals supports our inferences and conclusions. Further study of other diverse assemblages of Mesozoic mammaliaforms will allow the testing of whether our results are truly indicative of the macroevolutionary physiological status of Jurassic mammaliaforms or reflective of differing constraints on the particular populations studied. We further caution that our results should only be interpreted in terms of physiological evolution among Mammaliaformes and may not be indicative of broader macroevolutionary trends among fossil synapsids.

To conclude, the application of cementochronology, through SRCT imaging of fossil teeth from 10 mammaliaform taxa spanning the Jurassic adaptive radiation of mammals, has revealed significant differences in the physiological life histories of nonmammalian mammaliaforms, early crown mammals, and extant taxa. Patterns of growth rates, estimated using the radial width and texture of cementum increments, suggest that mammalian growth patterns first developed among early theriimorph crown mammals from slower patterns in nonmammalian mammaliaforms. These patterns correlate with differences in the relationship between body mass and BMR estimated from life span in these taxa, with only cladotherian mammals potentially within the BMR range of extant mammals. Differences in growth rates and patterns between extant and early crown mammals suggest that they also attained sexual maturity later in life and had yet to reach the metabolic levels of living mammals of similar size. We conclude that the mid-Jurassic radiation of crown mammals occurred coevally with the acquisition of the modern mammalian growth strategy. However, physiological evolution followed a long-fuse pattern, with considerable developments remaining between the earliest crown mammals and extant species.

## MATERIALS AND METHODS

### Fossil specimens

From a total sample of 582 specimens from 31 fossil clades imaged over seven synchrotron experiments, 219 specimens representing 21 clades were of sufficient preservational quality for counting cementum increments. Of this sample, 68 specimens representing 10 clades provided a minimum of three individuals identified at the genus level or five individuals identified to ordinal level of sufficient quality to study cementum (i.e., with sufficiently broad regions of AEFC cementum with no diagenetic alteration suitable for interpreting the biological cementum structure). See https://datadryad.org/stash/share/zZD__7SnbNJjS1t8M71VXBUv7USUr0yZgetr1BtpxvQ for examples of data for each fossil specimen studied.

Seventeen sampled *Morganucodon* and 16 *Kuehneotherium* specimens (from 206 SRCT scanned specimens of these taxa) originate from the Early Jurassic *Hirmeriella* fissure suite from Glamorgan, South Wales (United Kingdom) (*4*, *51*) and were accessed from the collections of the Natural History Museum, London (United Kingdom) (NHMUK) and the University Museum of Zoology Cambridge (United Kingdom) (CAMZM). All *Morganucodon* were from Pontalun 3 fissure and *Kuehneotherium* was from Pontalun 3 and Pant 2 and Pant 4 fissures (table S4). Specimens were originally prepared from the clay or marl matrix by immersion in either hot water or dilute acid [see (*4*, *52*)]. Of the total sampled specimens, 14 *Morganucodon* and 9 *Kuehneotherium* specimens were analyzed here.

Bathonian specimens studied originate from two assemblages and their respective formations. From 176 total SRCT scanned specimens, 7 specimens (three *Phascolotherium*, two dryolestoids, one *Krusatodon*, and one *Borealestes*) are from an excavation of the White Limestone Formation at Woodeaton Quarry (Oxfordshire, United Kingdom) by the NHMUK, prepared by bathing in water and buffered 7.5% formic acid, taxonomically identified, and accessioned at the NHMUK (*52*). Twenty-one specimens (six *Borealestes*, four dryolestoids, six *Krusatodon*, and five *Palaeoxonodon*) originate from Kirtlington Quarry exposure of the Forest Marble formation, excavated, and prepared by bathing in water and acetic acid, taxonomically identified, and accessioned to the Oxford University Museum of Natural History and NHMUK by Freeman (*53*) (table S5). Of the total sampled Bathonian specimens, five *Borealestes*, five dryolestoids, four *Krusatodon*, three *Palaeoxonodon*, and three *Phascolotherium* specimens were analyzed here.

Forty-one Guimarota fossils (17 *Dryolestes*, 13 *Haldanodon*, and 11 multituberculates) (from 267 total SRCT scanned specimens) originate from the Late Jurassic (Kimmeridgian ~157 to 152 Ma) Alcobaça Formation of Portugal, specifically from the Guimarota coal mine. The matrix was excavated, split, and examined for bones, then bathed in dilute potash lye, and taxonomically identified by the Free University of Berlin before being accessioned to the Museo Geológico of Lisbon, Portugal (denoted by “Gui Mam”) (*54*) (table S6). Of the total Guimarota sample, 13 *Dryolestes*, 9 *Haldanodon*, and 9 multituberculates were analyzed here.

The *Hirmeriella* and Guimarota samples comprise isolated postcanine teeth and dentaries with teeth in situ, but the Bathonian sample is composed entirely of isolated molariform teeth. Calibration of relative and absolute ages of eruption for molariform teeth performed for *Morganucodon* (*4*) and here for *Haldanodon* and *Dryolestes* (Supplementary Note 4; figs. S6 and S7) suggests that all molariform teeth erupted within the same (and most likely the first) year of life in all fossil taxa phylogenetically bracketed by *Haldanodon* and *Dryolestes* (Fig. 2). Sampling of *Kuehneotherium*, the only taxon without calibration of eruption ages and not bracketed by these two taxa, was restricted to anterior molar teeth to minimize the risk of misinterpreting the year of life represented by cementum growth layer groups.

### Extant specimens and data acquisition

Five wild European hedgehog (*Erinaceus europaeus*) individuals were provided for research from the Hedgehog Rescue (South Gloucestershire, United Kingdom); all of which had deceased under their care following injury. Ten Rhesus macaque (*Macaca mulatta*) individuals were provided by the Primate Breeding Facility of Public Health England (Salisbury, United Kingdom) after being humanely euthanized [not for the purpose of this experiment; see (*27*)]. Permanent lower m1 teeth (herein lm1) were removed from each individual and prepared for SRCT scanning by removing the tooth roots from the crown, following the protocol outlined by Newham *et al.* (*27*).

Three field vole (*Microtus agrestis*) and three field mouse (*Mus musculus*) dentary specimens were isolated from owl pellets from the collections of the University of Bristol Biological Sciences and taxonomically identified by E.N. The lm1 teeth were removed and used to create ~90-μm-thin sections following the protocol outlined by Newham *et al.* (*27*). These were imaged using reflected light microscopy via a Nikon Eclipse Lv100 microscope using a 50× objective, equipped with a Nikon DS-Fi2 digital viewfinder under polarized and nonpolarized light.

Data for all other extant taxa were taken directly from the literature or were provided upon request in the form of digital micrographs of cementum thin sections (table S1). While data on the sex of individuals were provided for certain samples, most data provided did not include this, and as the same data could not be estimated for our fossil sample, individuals were not subsampled based on sex for this study.

### X-ray computed tomography

*Hirmeriella* samples (87 *Morganucodon* and 119 *Kuehneotherium*) and five *E. europaeus* specimens were mounted to 2-mm-wide carbon fiber rods using paraloid glue and scanned during two successive SRCT experiments at the ID19 beamline of the European Synchrotron Radiation Facility, Grenoble, France (proposal ES 152; 18/04/2014-22/04/2014) and the TOMCAT beamline of the Swiss Light Source, Villigen, Switzerland (proposal 20141278; 13/04/15-16/04/15). Voxel sizes for those experiments were 347 and 325 nm [see (*4*) for full synchrotron scanning and experimental settings].

Ten *M. mulatta* specimens were mounted on carbon fiber rods using cyanoacrylate glue and scanned during a 3-day experiment at the TOMCAT beamline (proposal 20151391; 04/03/16-07/03/16). A quasi-monochromatic beam was set to an energy of 21 keV using a double multilayer monochromator, a 20-μm-thick LuAG:Ce scintillator, and a pco.edge 5.5 detector. Specimens were scanned using single propagation distance tomography (14-mm sample-to-detector distance), an exposure time of 150 ms, and either 1500 angular projections over a 180° angular range or 3000 angular projections over a 360° range at a voxel size of 660 nm.

A total of 108 Bathonian specimens and 4 Guimarota specimens were mounted on carbon fiber rods using paraloid glue and scanned during a 3-day experiment at the TOMCAT beamline (proposal 20160404; 18/11/16-21/11/16). A quasi-monochromatic beam was set to an energy of 20 keV using a double multilayer monochromator, an LSO:Tb scintillator, and a pco.edge 5.5 detector. Specimens were scanned using single propagation distance tomography (14-mm sample-to-detector distance), an exposure period of 150 ms, and either 1500 angular projections over a 180° angular range or 3000 angular projections over a 360° range at a voxel size of 330 nm.

A total of 68 Bathonian specimens and 7 Guimarota specimens were mounted on carbon fiber rods using paraloid glue and scanned during a 4-day experiment at the ID19 beamline (proposal ES-502; 01/02/17-05/02/17). A single harmonic U13 undulator delivered a pink beam with energy centered on ~26 keV (with a 1.4-mm Al filter used to minimize lower energies) using a 10-μm-thick GGG:Eu scintillator and a pco.edge 5.5 detector. Scans used single propagation distance tomography (15-mm sample-to-detector distance), an exposure time of 250 to 300 ms, and 1500 angular projections over 180° with a voxel size of 347 nm.

Last, 77 Guimarota specimens were mounted within foam and scanned during a 3-day experiment at the ID19 beamline (proposal ES-583; 14/02/18-17/02/2018), and 179 Guimarota tooth specimens were mounted on carbon fiber rods using water-based glue and scanned during a 3-day experiment at the TOMCAT beamline (proposal 20180876; 13/09/18-16/09/18) using the respective experimental settings described above. See table S7 for a summary of all taxa scanned in each experiment.

All specimens were SRCT scanned within the coronal third of the tooth roots, which is the region considered to consist of predominantly AEFC. AEFC [as opposed to cellular intrinsic fiber cementum (CIFC)] is the cementum tissue type known to be deposited consistently through life with the most robust circum-annual periodicity. All scans were reconstructed using a standard filtered back-projection algorithm using the PyHST2 (ID19) or “Gridrec” software package (TOMCAT) coupled with a “Paganin-style” single-distance phase retrieval algorithm (*55*) that is integrated and available at the respective beamlines (*56*, *57*). For phase retrieval of TOMCAT data, a decrement of the real part of the refractive index β = 1.7 × 10^−10^ and an imaginary part of the refractive index δ = 3.7 × 10^−8^ have been chosen and β = 9.8 × 10^−9^, δ = 8.1 × 10^−8^ for data generated at ID19 (see Supplementary Note 3 for analysis and discussion of the effects of differing beamline optics, experimental settings, and preprocessing steps on analyses performed here).

### Increment width measurement

Volumetric SRCT datasets with submicrometer voxel sizes present an exponential increase in data available per specimen compared to the limited number of 2D thin sections that can be created for millimeter-scale fossil teeth. This increase can offer a significant improvement in the accuracy of increment counts (*4*, *30*) and representation of a specimen’s increment patterns. However, this, in turn, requires a robust methodology for analyzing and comparing increment counts and patterns in volumetric datasets, which balances accuracy against efficiency of analysis. Here, we present, validate, and optimize a procedure for measuring and comparing patterns of increment growth in fossil cementum in an optimum number of locations within SRCT datasets to provide a robust estimate of growth patterns that are representative of the entire tooth root (i.e., no significant change in the SD of increment measurements when more than this number of locations is measured).

Of the 582 specimens, from 31 fossil clades imaged over seven synchrotron experiments, 219 fossil SRCT datasets were selected based on the preservation of cementum increments following qualitative analysis in ImageJ/Fiji (*58*). Specimens were subsampled into three distinct categories based on preservational quality, increment image contrast, and proportion of SRCT artifacts (Supplementary Note 2; figs. S1 to S3). Category “a” specimens showed highly contrasting increments with no significant chemical and/or physical diagenesis affecting their patterns and no interruption of patterns by SRCT imaging artifacts. These were used in all analyses performed here. Category “b” specimens showed less highly contrasting increments than category a specimens but no significant disturbances by diagenesis or artifacts and were only subjected to increment width analysis. Category “c” specimens showed clear chemical and/or physical diagenesis, and/or incremental patterns were overprinted or interfered with by SRCT imaging artifacts. These were not used in analyses here. See https://datadryad.org/stash/share/zZD__7SnbNJjS1t8M71VXBUv7USUr0yZgetr1BtpxvQ for examples of data for each fossil specimen studied presenting the full range of preservational quality.

“Virtual thin sections” [VTS; see (*4*)] were then created for the entire data volume for each of category a and b specimens by using the *z*-projection technique to combine the information in 10 transverse slices in Image J/Fiji and saved as “stacks” of 8-bit TIFFs (Tag Imaging File Format). In addition to standardizing the region of the tooth root sampled for SRCT scanning, the region within each scan chosen for analysis was also standardized to minimize the risk of comparing differing cementum tissues under differing developmental pressures. Comparison between histological data and SRCT data of the same regions of extant mammal cementum by Newham (*59*) and Newham *et al.* (*27*) suggests that AEFC can be isolated from CIFC in SRCT data through the detection of cellular voids in CIFC. While AEFC is known to contain occasional cellular voids (*60*), they are significantly more concentrated in CIFC, and incrementation found in AEFC is distorted and/or disturbed in CIFC (fig. S4). Cementum data were then isolated and straightened from regions of interest (ROIs) that contained no cellular voids (evidence of CIFC) and avoided regions of anomalous cementum thickness (i.e., evidence of angular drift in cementum development rates due to occlusal forcing) following Newham *et al.* (*27*). Increments were studied in straightened cementum data by measuring grayscale distributions in micrometers along 10-pixel thick radial transects through the cementum, between the hyaline layer of Hopewell-Smith, and the outer cementum boundary (Fig. 1). Increment widths along these transects were analyzed by measuring the distances between subsequent peaks in grayscale, designated as the center points of circum-annual light increments, to provide growth layer group measurements for each year of life. The last year of life was not included as it is not possible to assess if this represents a complete year of growth, and this is often also obscured by a pronounced phase fringe due to the shift in the x-ray phase at the cementum-air boundary (Fig. 1). While phase shifts also have the potential to create light/dark features within the cementum that are not analogous to growth increments (*60*, *61*), our previous comparisons performed between SRCT data and histological data for the same scanned regions in (*36*) [figure 7 in (*36*); fig. S5] suggest that increments seen in SRCT data represent optical increments in histological data.

This method was applied to a subsample of fossil data to validate an optimal number of transects to study per SRCT volume (Supplementary Note 4). Following this optimization, growth layer group widths were measured over eight evenly spaced radial transects in each of nine evenly spaced VTS in every SRCT dataset selected for analysis (including *E. europaeus* and *M. mulatta*). Selection was based on a qualitative assessment of cementum preservation through the entire scanned volume for each specimen. Only teeth with sufficient proportions of cementum, with no diagenetic fabric or taphonomic damage [see supplementary figure 2 in (*4*)] for evenly spaced sampling through the entire volume, were considered for analysis here.

All studied taxa are considered as diphyodont (undergoing a single replacement of teeth) on the basis of previous studies of representative dentulous specimens and/or phylogenetic bracketing (*4*, *19*, *21*, *50*, *62*). Although additional replacement of certain teeth is known in some eutriconodontan gobiconodontids (*63*–*65*), it has been shown that this is not a generalizable pattern within eutriconodontans (*66*). Our sample includes the “amphilestid” eutriconodontan *Phascolotherium*, but gobiconodontids were not represented in our sample. Cementum increments imaged in our fossil sample were thus considered as representative of the entire life history of each specimen (excluding any portions of the cementum taphonomically removed) following eruption of the respective tooth. This understanding, alongside the findings here (Supplementary Note 4) and in a previous study (*4*) of diphyodont tooth replacement occurring within the first 2 years of life, also allowed any teeth with increment counts higher than 2 years to be interpreted as permanent teeth.

### Cementum texture analysis

Analysis followed Newham *et al.* (*27*), where texture was defined as the spatial organization of grayscale values within samples of cementum data using this distribution as a third dimension/*z* axis (Fig. 1J and fig. S1). Twenty-one 3D surface profiling measures, previously used to define and compare biological surfaces (*27*, *67*), were applied to the SRCT and histological data used in other analyses (see Supplementary Note 1 and table S3 for description of each measure). Data for eight extant taxa (table S2) were subsampled into juvenile and adult portions based on the known mean age at eruption and replacement of the respective tooth and known mean age at sexual maturity. Subsample size (in pixels) was determined by the shortest radial length of either juvenile or adult portions of the particular dataset (excluding the hyaline layer of Hopewell-Smith for juvenile data and the last year of growth for adult data), which was used as the radial length (when multiplied by the respective voxel size) for both subsamples. To ensure that equally sized juvenile and adult portions of data were sampled, the subsample width was either double this length or as close to this value as possible if the dataset size was too small.

Subsamples were analyzed using a custom-designed algorithm to use each measure in the MATLAB statistical environment (R2021a; The MathWorks Inc., Natick, MA, United States), validated for SRCT (*27*) and histological (*59*) cementum data. Outputs were compared between juvenile and adult data for each extant dataset using ANOVA (table S3), and the 14 measures with significant differences for every dataset were subjected to PCA (Fig. 4).

### Fossil body mass estimation

Body masses estimates for *Morganucodon* (17.9 g) and *Kuehneotherium* (23.8 g) follow Newham *et al.* (*4*) based on measured and estimated skull length and dentary length. The estimate for *Palaeoxonodon* is based on dentary length (*68*), following the regression published by Foster (*69*). Estimates for *Dryolestes* and *Haldanodon* are from Kirk *et al.* (*70*). The estimate for Guimarota multituberculates (45 g) is an averaged value from estimates for three Guimarota taxa (41 to 48 g) in (*47*).

Because of a lack of diagnostic cranial and dentary material for most taxa [excluding *Borealestes* (*71*)], body masses for taxa from the Forest Marble sample were based on a regression equation developed in (*59*). The dentary length was estimated by developing a series of scaling relationships between mesial/distal lm1 length and dentary length for 39 fossil taxa of differing molar counts (not including multituberculate or haramiyid taxa) from the literature (table S9). Expected molar counts were generated for each taxon either from direct counts, estimates from the literature, or expected counts based on their phylogenetic affinity. A series of scaling factors was then generated by using linear least-squares regression to compare the lm1 length to dentary length in increasingly broad samples of molar counts (table S10). The broadest (highest *n* value) subsample that included the relevant molar count for each Kirtlington docodont, while providing the highest value for the coefficient of determination (*r*^2^) value (0.84), comprised 29 taxa of known dentary length and molar counts between 3 and 5. The regression equation between the lm1 length and dentary length in this subsample followed

dentary length (mm)=[lm1 length (mm) * 10.184]+8.026

The resultant dentary length estimates, based on the lm1 length for the Bathonian docodont taxa, were then used to estimate their body mass in grams following the formula of Foster (*69*) [see (*1*)]. The precision of the estimate for *Borealestes* using this method (32 g) versus that estimated using the dentary presented by Panciroli *et al.* (*71*) (36 g) suggests that the body mass estimates provided for docodonts here are reliable relative to other methods.

### Life-span and BMR estimation

Life-span estimation was based on counts of growth layer groups by E.N. in the ROIs used for increment width measurements. Following Newham *et al.* (*36*), grayscale transects were split into five segments of equal length, and the mean and SD of each segment were calculated and plotted (fig. S1). Growth layer group counts were based on the number of grayscale peaks (i.e., light increments; Fig. 1) that exceeded the upper SD of the respective segment. These were, in turn, used to estimate the mass-specific standard metabolic rate (msSMR) [synonymous with the msBMR in mammals (*10*)] using the regression formulas between the two variables, created using PGLS regression of their log-transformed values, for extant mammals and non-avian reptiles, respectively, from Newham *et al.* (*4*) (fig. S3)

$${\text{log}}_{10}\;\text{mammal msSMR}=-0.237\left[{\text{log}}_{10}\;\text{life span}\;\left(\text{years}\right)\right]-0.083$$

$${\text{log}}_{10}\;\text{reptile msSMR}=-0.83\left({\text{log}}_{10}\;\text{life span}\right)-0.31$$

### Mass-specific cementum growth rate

Mean increment thickness positively correlates with body mass in extant mammals (fig. S2). This correlation must be accounted for when comparing the growth rates of extant and fossil mammals of differing body size. The correlation was here accounted for by using phylogenetically informed nonlinear regression (PGNLR) (*37*) to compare log-transformed mean body mass (g) and log-transformed mean width of first recorded cementum growth layer group (μm) for each extant taxon studied here (*n* = 21). The first recorded growth layer group was chosen as this most reliably represents the maximum growth rate of the respective taxon and is least likely to be biased by the pattern of growth rate reduction experienced by extant mammals. Body masses were obtained from an online Ecological Archives database (http://esapubs.org/archive/ecol/E084/094/metadata.htm) and the AnAge database (https://genomics.senescence.info/species/).

PGNLR was performed in the “R” statistical environment (version 4.3.2) with the “minpack.lm,” “ggplot2,” “gridExtra,” “phytools,” and “dplyr” packages installed. A phylogenetic subset of the extant mammals sampled was downloaded from https://vertlife.org (data S1) representing their phylogenetic relationships. This was inputted into a nonlinear (exponential) regression model between log-transformed body mass and log-transformed mean first increment width using the methodology of Jhwueng and Wang (*37*). Four phylogenetically informed models were compared using the approximate Bayesian computation: an exponential model using Brownian motion for phylogenetic trait evolution (OUGBM), an exponential model using the Ornstein-Uhlenbeck process for phylogenetic trait evolution (OUGOU), a linear model using Brownian motion (OUBM), and a linear model using the Ornstein-Uhlenbeck process (OUOU). Model performance was compared using the posterior probability (*P*) from 500 repeats for each model. From this comparison, the OUGBM model was selected (*P* = 0.0015), providing a regression equation of

$$\begin{array}{c} \text{logged first growth layer group width}\;(\mu m)=\\ 0.270*\text{exp}\left[0.340*\text{logged mean body mass}\;\left(g\right)\right]+0.564 \end{array}$$

This was used to correct measured growth layer group widths and create an msGR measure for each year of life based on mean width measurements for each extant and fossil clade.

### Modeling of growth patterns

Patterns in the mean msGR for each year of life for fossil and extant taxa were modeled by regressing against year of life for 10 nonlinear models using the PAST (version 4.07b) statistics software (*72*) (table S11). Fit of each model to the data was assessed using their Akaike information criterion (AIC) and *r*^2^. The best fitting model for each taxon (the model with the lowest AIC and highest *r*^2^) was used to abstract its overall trends in growth rate through life.

### Age at sexual maturity data and analysis

Information on mean body mass and mean age at sexual maturity was obtained for 776 extant mammals (mean body mass = 746.6 kg, SD = 7095.22 kg; mean age at sexual maturity = 1.91 years, SD = 2.16 years) from the dataset of Herculano-Houzel (*15*). Information on mean body mass and mean age at sexual maturity was obtained for 411 non-avian reptiles (mean body mass = 1.1 kg, SD = 6.14 kg; mean age at sexual maturity = 2.08 years, SD = 1.67 years) from the dataset of Scharf *et al.* (*38*). These measures were compared using phylogenetically informed ANCOVA following Smaers and Rohlf (*73*) using the “gls.anvoa” function in R with the “ape,” “geiger,” “nlme,” and “phytools” packages installed. The time-calibrated combined phylogenetic tree created by Newham *et al.* (*4*) was used to classify data for the respective clades into two factorial variables (“reptile” and “mammal”), and the phylogenetic influence of these variables was assessed following the method outlined by Newham *et al.* (*4*).

### Statistics

Exponential PGLS regression of the mean mammal body mass against the mean width of first cementum increment: log_10_ first growth layer group width (μm) = 0.270*exp[0.340*log_10_ mean body mass (g)] + 0.564; 95% confidence interval (CI) = 0.192; *r*^2^ = 0.930, *P* < 0.001. One-way ANOVA comparison of pooled msGRs between fossil crown mammals and nonmammalian mammaliaforms; Shapiro-Wilks normality test: fossil crown mammals *W* = 0.954, *P* = 0.206, nonmammalian mammaliaforms *W* = 0.987, *P* = 0.832; test statistics *F* = 91.85, df = 69, Cohen’s effect size *d* = 2.309, *P* < 0.001. One-way ANOVA comparison of pooled msGRs between the first 6 years of life in fossil crown mammals and nonmammalian mammaliaforms; Shapiro-Wilks normality test: fossil crown mammals *W* = 0.929, *P* = 0.1053, nonmammalian mammaliaforms *W* = 0.977, *P* = 0.762; test statistics *F* = 75.12, df = 51, Cohen’s effect size *d* = 2.424, *P* < 0.001. One-way ANOVA comparison of pooled msGRs between extant and fossil mammals; Shapiro-Wilks normality test: extant mammals *W* = 0.9692, *P* = 0.015, fossil mammals *W* = 0.954, *P* = 0.204; test statistics *F* = 44.55, df = 117, Cohen’s effect size *d* = 1.395, *P* < 0.001. One-way ANOVA comparison of pooled msGRs between the first 2 years of life and the succeeding years of life for Mesozoic nonmammalian mammaliaforms; Shapiro-Wilks normality test: first 2 years of life *W* = 0.851, *P* = 0.06, succeeding years of life *W* = 0.966, *P* = 0.439; test statistics *F* = 11.89, df = 38, Cohen’s effect size *d* = 1.396, *P* = 0.001. PGLS regression of the mean mammal body mass against the maximum wild life span: log_10_ life span = 0.26(log_10_ body mass) + 0.16; 95% CI = 0.05; *r*^2^ = 0.69, *P* < 0.001. PGLS regression of mean non-avian reptile body mass against maximum wild life span: log_10_ life span = 0.26(log_10_ body mass) + 0.60; 95% CI = 0.08); *r*^2^ = 0.46, *P* < 0.001. PGLS regression of mammalian life span against msSMR: log_10_ msSMR = −0.237(log_10_ life span) − 0.083; 95% CI = 0.07; *r*^2^ = 0.59, *P* < 0.001. PGLS regression of reptilian life span against msSMR: log_10_ msSMR = −0.83(log_10_ life span) − 0.31; 95% CI = 0.255; *r*^2^ = 0.43, *P* < 0.01. Phylogenetic ANCOVA comparison of PGLS regression slopes for the mean body mass against age at sexual maturity in extant mammals [log_10_ age at sexual maturity = 0.164(log_10_ body mass) − 0.483; 95% CI = 0.033; *r*^2^ = 0.52] and extant non-avian reptiles [log_10_ age at sexual maturity = 0.170(log_10_ body mass) + 0.102; 95% CI = 0.031; *r*^2^ = 0.252]; slopes are not statistically different (*F* = 1.822, *P* = 0.320), while means are significantly separated [*F* = 3.444, df = 1186, partial eta squared (η*^2^p*) effect size = 0.32, *P* = 0.0326].

## Supplementary Materials

### This PDF file includes:

Supplementary Text Figs. S1 to S14 Tables S1 to S11 Legend for data S1 References
