# Supplementary material for: The origins of mammal growth patterns during the Jurassic mammalian radiation
Source: Sci Adv. 2024 Aug 7;10(32):eado4555. doi: 10.1126/sciadv.ado4555 (PMC11758522; doi:10.1126/sciadv.ado4555)
Supplement: Supplementary file 1 — Supplementary Text Figs. S1 to S14 Tables S1 to S11 Legend for data S1 References [file sciadv.ado4555_sm.pdf]

Science Advances
AAAS

Supplementary Materials for

The origins of mammal growth patterns during the Jurassic mammalian radiation

Elis Newham *et al.*

Corresponding author: Elis Newham, elis.newham@googlemail.com; Pamela G. Gill, pam.gill@bristol.ac.uk

Sci. Adv. **10**, eado4555 (2024)
DOI: 10.1126/sciadv.ado4555

The PDF file includes:

Supplementary Text
Figs. S1 to S14
Tables S1 to S11
Legend for data S1
References

Other Supplementary Material for this manuscript includes the following:

Data S1

Supplementary Text

Supplementary Note 1: Discussion and comparison of the paleobiogeography of fossil samples.

Aside from significantly different time intervals, our three fossil samples originate from disparate localities in terms of latitude, longitude, geography, and climate. These factors must be considered in terms of their effect on the biology and ecology of their mammal faunas, and sampling of their growth patterns. For instance, cementum increments in living mammals have been shown to follow localized climate patterns, with rainfall patterns in equatorial regions creating a “doubling” effect of two dark increments per-year as the effects of these patterns outweigh their weak circum-annual cyclicity (*74*). The most reliable circum-annual patterns are generally found for mammals in temperate latitudes that experience strong circum-annual seasonality (*22*).

All three fossil samples originate from temperate–to–sub-tropical paleolatitudes, with minimal risk of the “doubling” effect biasing counts and observed patterns of cementum increments. The Early Jurassic *Hirmeriella* fissure fauna was approximately 30° North as part of an Island archipelago that experienced seasonal climates (*33*). The Mid Jurassic Forest Marble fauna originates from a slightly higher latitude (~35° North), and also from a high energy coastal dune environment with frequent seasonal storms (*34*). The Guimarota fauna originates from ~30° North, and is suggested to be part of a temperate–to–sub-tropical archipelago system (*75*).

However, the taxonomic diversity, lithology, and taphonomy of fossils from each fauna indicates differing environmental and ecological influences in our sampled faunas, which may either directly or indirectly affect their demographic composition and observed

growth patterns. The *Hirmeriella* sample is comprised of two primary faunas representing slightly differing time periods, environments and ecologies (*33*). The older fauna is taxonomically depauperate, with very little evidence of complex trophic pyramids and predation, and limited food resources indicated by low floral diversity and dominance (>95%) of *Hirmeriella* (*76*). The younger fauna is significantly more diverse, with the first evidence of herbivorous taxa. However, there continued to be very little evidence of predators in significant portions (*33,76*). This is suggested to be due to a rise in sea-level through the Rhaetian marine transgression and in-turn a rise in local water tables and improved access to fresh water. Both faunas provide evidence for strong seasonal cyclicity, including seasonal storms and fires. In lieu of predation as the origin for mammal material (rejected due to lack of evidence of acid digestion, or concentration of predatory fossils), it has been suggested that seasonal wildfires are the primary origin of material as catastrophic killing events (*33*). The *Morganucodon* sample is all from one fissure in the older depauperate fauna, and the *Kuehneotherium* sample is taken from both faunas.

The Mid Jurassic Forest Marble fauna represents a coastal dune ecosystem and displays evidence of relatively high environmental productivity and turnover (*34*). High predation pressure is suggested from a diverse array of predatory taxa present (including theropods, pterosaurs, and freshwater crocodiles), and densely populated, diverse, and fragmentary assemblages of microvertebrates interpreted as originating from predatory middens (*34,52*). Resource competition and partitioning is also suggested from the relatively high taxonomic diversity, representing taxa in the Forest Marble sample sharing and competing for specific ecological niches. While strong seasonality and seasonal storms have been suggested, the Forest Marble experienced less drastic environmental change compared to the *Hirmeriella* fauna, through its depositional timeframe.

The Late Jurassic Guimarota mammal fauna is taxonomically depauperate relative to the Forest Marble, with the majority of its considerable quantity and concentration of material represented by four genera (*35*). The relative completeness of this material also suggests a relatively stable, low energy environment with little post-depositional transportation of material. The fauna has yet to produce the diverse vertebrate assemblage (including several mammal taxa) commonly found in the local contemporaneous Leiria and Lisboa formations, which has been interpreted to be due to a smaller catchment area and reduced ecological opportunities in the Guimarota (*35*). The surrounding Lourinhã formation provides evidence for high seasonal precipitation, with the Guimarota environment forming at low altitudes under significant standing water (*75*). It is thus suggested that the Guimarota biome existed under localized swamp conditions, with low productivity and little resources to sustain a diverse mammalian assemblage.

These environmental differences may be reflected in the results of our analyses, either through direct effects on the life histories of mammals studied, or by creating sampling biases. The Forest Marble data shows a marked disparity between the estimated lifespans of docodontan mammaliaforms and all sampled crown mammals (Figs 2, 3a). This is in direct contrast with the lifespans estimated for crown mammals in the Guimarota sample, that are comparable to docodontans and other mammaliaforms (Figs 2-3). Behavioral plasticity observed at species and population-level (*77*) has been frequently observed for living vertebrates in response to disparities in environmental factors including resource availability, competition, and predation pressure (*78*). Predation is known to have a complex range of impacts on the age structure of prey populations, based on a number of factors beyond the detection limits of this study (e.g. age selection, sex selection, predatory strategies). However, the growth patterns of Forest Marble crown mammals provided here suggest that they are predominantly sub-adult individuals (i.e. below or at the point of truncation in growth-rate –

Fig. 2) that did not survive significantly past sexual maturity. If the predominant origin for Forest Marble fossils is from predatory middens, this may suggest a sampling bias due to selective predation of younger individuals.

Predation of Forest Marble crown mammals does not fully explain the disparity of lifespans found between them and cohabiting docodontans, when members of the same crown clades show equally long lifespans to docodontans in the Guimarota sample. The "Pace of Life Syndrome" (POLS) hypothesis (*79*) suggests that a feedback system operates between resource-driven behavior and physiological life history, with correlations found between resource-driven behavioral traits and metabolic physiology (*80-82*), survival and predation (*83-85*) and age at maturity (*50,86,87*). This places the feedback between physiology and behavior, with increasing metabolic rates necessitating greater resource acquisition and permitting different behavioral strategies, in the context of the environment of a particular species or population. High productivity environments (e.g. the Forest Marble biome) that promote behaviors necessitated/permitted by an organism's metabolism for increased acquisition of resources, at the expense of survival and risk of predation, may amplify existing differences in the life histories of cohabiting species with different metabolic physiologies. Whereas those that provide less benefit to resource acquisition behaviors (e.g. the Guimarota biome), may instead reduce life history differences between cohabiting species.

Evidence for these contrasting POLS regimes in the Forest Marble and Guimarota samples from our data includes differing age-at-sexual maturity estimates for cladotherian-grade crown mammals, with growth-rates of both Forest Marble taxa truncating earlier in life (Fig. S5). The Guimarota taxa *Dryolestes* and *Haldanodon* also exhibit slower mass-specific growth-rates than phylogenetically close and less derived Forest Marble taxa. These results

suggest a comparatively slower pace of life in the Guimarota biome, potentially reflective of its lower productivity.

Supplementary Note 2: preservational categorization

Preservational quality of fossilized cementum shows significant variation between specimens (online supporting data) (Newham et al. (*4*) Fig. S13). As previously noted (*4*,*39*), several diagenetic fabrics can be detected in different specimens that indicate chemical and/or physical disturbances to cementum incrementation including cracking or damage to the cementum tissue, combined with inherent natural variation in increment quality and contrast. Fossilization has also been shown to preserve radial bundles of Sharpey's fibers with comparable contrast in SRCT data to circumferential cementum increments (*4*). SRCT imaging also provides a series of artefacts including ring artefacts (concentric circular features of differing grey scale centered at the centerpoint of the respective SRCT slice) that can obscure cementum increments if closely aligned. These have the potential to inhibit the use of certain specimens for specific analyzes performed in this study, and necessitated the subsampling of the total fossil specimen into preservational categories.

Three categories were distinguished here. Category "a" consists of the best preserved specimens, with regions of interest (ROIs) comprising undisturbed cementum with highly contrasting increments and no evident chemical/physical diagenesis (Fig. S12). If ring artefacts are present, they do not obscure cementum increments. These specimens were used for measuring increment widths and msGRs, and in cementum texture analyzes. Category "b" consists of specimens with ROIs comprising cementum with clear increments and no significant diagenetic alteration or physical damage to the cementum, but lower increment contrast than category "a" specimens (and higher proportionate contrast of Sharpey's fiber bundles relative to increment contrast in certain specimens – e.g. *Borealestes* specimen

NHMUK PV M 100072) (Fig. S13). These specimens were only used in increment width and msGR analysis. Category "c" consists of specimens with ROIs that show evident chemical diagenetic fabrics and/or physical damage to the cementum tissue that obscure increments (Fig. S14). These specimens were not included in analyzes.

Supplementary Note 3: Description of 3D surface profiling measures and cementum "texture" analysis.

The structure and growth of cementum during juvenile and adult life phases was here compared by using the spatial organization of greyscale values in SRCT and 8-bit histological microphotographs as a 'third dimension' for which to employ 21 3D surface profiling measures, commonly used to profile and compare surfaces in tribology and biology (*67*). These comprise height measures (the distribution of greyscale values above/below the average value for the surface), spatial measures (the isotropy, directionality and heterogeneity of greyscale values across an image x/y axes), hybrid measures (combining x/y axes information with the 'z axis' provided by greyscale distribution), and 'functional' measures (that characterize volumetric information based on the material ratio of the respective surface: the ratio between the surface-bearing area and cross-sectional area) described in Supplementary Table 3. In order to compare results of these measures between SRCT and histological data, the spatial resolution and fidelity of SRCT imaging for capturing increments and other details of cementum found in thin-section histology has been validated by Newham et al. (*36*), and the precision between measurements made in SRCT and thin-section data of the same regions validated in Newham (*59*).

Twenty-one 3D measures previously validated for studying grayscale "texture" (grayscale variation due to structural differences) in SRCT and histological cementum data (*27,39*) (Fig. 1; Fig. S1) were applied to equally sized areas of cementum deposited before

and after the inflection point in msGR, in the same extant data analyzed for msGR patterns (Fig. S1; Supplementary Table 3). ANOVA analysis of the resulting data revealed 14 measures with significant differences between juvenile and adult data for every taxon (Supplementary Table 3).

Of the 21 measures applied to the full sample of cementum data, 14 provided significant differences between sampled juvenile and adult cementum data when compared using ANOVA (Supplementary Table 3). When these results were subjected to principal components analysis (PCA), loading of Principal Component 1 primarily comprised of height and spatial measures, with positive values reflecting greater contrast and increased anisotropy, while loading of Principal Component 2 primarily comprised of functional measures, with positive values showing greater greyscale variation within the standard deviation of the sample.

Resampling from original 16-bit to 8-bit was performed to improve computational time and decrease computational loads, allowing texture measures to be developed, tested and performed on commonly available laptop computers. However, this resampling presents a significant decrease in absolute greyscale contrast from 65,536 values to 256 values. This may in-turn alter observed differences between samples of cementum data and must therefore be accounted for when interpreting biological differences in cementum structure using our methodology. We here validated the effects of subsampling to 8-bit on our texture measures by using them to analyze the original 16-bit data for subsamples of fossil mammals, and compare the texture space occupation to those of the primary 8-bit sample (Fig. S9). Comparisons show comparable patterns in texture space occupation and overlap for both bit-rates in each fossil mammal sample: non-mammalian mammaliaforms show considerable overlap between juvenile and adult cementum; theriiform fossil mammals (*Dryolestes* and

Late Jurassic multituberculates) show discrete texture space occupation for juvenile versus adult cementum.

A validation test was also performed to characterize the effects on applied texture measures from differing experimental settings, beamline optics, and preprocessing steps at the TOMCAT beamline (Swiss Light Source) versus the ID19 beamline (European Synchrotron Radiation Facility) (Materials and Methods). These may impart differences in the distribution of absolute greyscale values in the data acquired at each beamline, which may in-turn create non-biological differences in greyscale texture in cementum data. The data used for texture analysis originates from the same respective beamline for all studied taxa except for *Krusatodon.* So the potential effects stemming from data acquisition and treatment at each beamline only applies for this taxon. These effects were tested for here by applying PCA to compare the results of texture measures between datasets acquired at each beamline (Fig. S10a). The resultant PCA plot shows significant overlap between data acquired at each beamline, suggesting that the influence of differing origin is not significant enough to effect observed patterns in cementum texture.

As a final test for the effects of data origin on observed patterns in cementum texture, an unsharp mask (2 pixel radius; 0.6 weight) was applied in ImageJ/Fiji to alter greyscale distribution in a controlled manner for data in subsamples from *Dryolestes* and *Morganucodon* - fossil taxa with contrasting patterns in observed texture measures between juvenile and adult cementum data (Fig. 4). Texture measures were applied to data processed with this unsharp mask, and results compared with those of the original data using PCA. Unsharp-processed data overlaps with original data in each subsample (Fig. S10b-c). For *Morganucodon*, all data overlaps with no distinct ‘texture-space’ occupation between ‘juvenile’ and ‘adult’ data (both original and unsharp-processed). For *Dryolestes*, unsharp-

processed data overlaps with original data, with juvenile and adult data separated along PC1 into separate ‘texture spaces’.

Supplementary Note 4: Calibration of age-at-eruption for Jurassic fossil taxa.

The study of isolated teeth may risk misinterpreting the year-of-life represented by cementum increments in respective taxa if the age at-which different teeth erupt differs by more than one year. To assess this risk, we compared previous estimates of age-at-eruption for different tooth types in *Morganucodon* (Newham et al. (*4*)) with pilot data for the Guimarota taxa *Dryolestes* and *Haldanodon* (Figs S6-S7). *Morganucodon*, the most stem-ward taxon in our fossil sample, shows evidence for a potential difference of up to one year between the eruption of lp1-lm2 teeth in its first year-of-life and the eruption of lm3 (and li4) in its second year-of-life. For *Dryolestes*, one of our most derived fossil taxa, the same counts were found for cementum growth-layer-groups in each of the eight molariform teeth in the dentary of a well-preserved dentulous specimen (Fig. S7), suggests that each of its eight molariform teeth erupt within the same year-of-life. Comparing these counts to those of lines-of-arrested growth (LAGs) in the dentary of the same specimen suggests that eruption occurred within the first year of life. While suitable dentary LAGs could not be identified in a dentulous specimen of *Haldanodon*, the finding of the same cementum growth-layer-group counts between the lm1-lm5 teeth of a well-preserved dentulous specimen also suggests that these tooth types erupted within the same year-of-life (Fig. S8). LAGs in the dentary bone have been used for age determination in several small-bodied extant mammal taxa (*22,88,89*), although some validation studies have questioned their accuracy (*90*). Phylogenetic bracketing of other studied taxa between *Haldanodon* and *Dryolestes* (Fig. 2) suggests that their molariform teeth also erupted within the same year-of-life. Sampling of the only taxon without calibration of eruption ages and not bracketed by these two taxa, *Kuehneotherium*,

was restricted to anterior molariform teeth in order to minimize the risk of misinterpreting the year-of-life represented by cementum growth-layer-groups.

Supplementary Note 5: Validation and optimization of volumetric subsampling for SRCT cementum data.

A robust methodology for subsampling cementum data from volumetric SRCT datasets was created, validated, and optimized here by testing the effect of sampling density on the variation of cementum increment width measurements in a subsample of five fossil specimens (Supplementary Table 9). Sampling density was tested by comparing the standard deviations of width measurements taken from increasing numbers of radial transects measured per-virtual thin section (VTS) (Materials and Methods), and increasing numbers of VTS per-specimen.

In a subsample of single straightened VTSs for five fossil specimens, increments were measured along 13 10-pixel-thick transects drawn iteratively from each endpoint of the straightened region of interest (so that measurements sampled the entire ROI independently of the number of transects taken) (13 transects = 130 μm was the minimum width of whole ROIs in the selected subsample). For each sequentially studied transect, GLG width measurements were compiled and the moving mean value and standard deviation (SD) calculated and logged. The optimum number of transects studied per-VTS was defined as the minimum number of measurements required to provide a consistent SD with increasing transect count (Fig. S11). This was determined by regressing the number of measurements against SD and assessing the number of transects at which the regression between these two variables flattened ($-0.01 <$ change in SD/ increase in transects < 0.01). This occurred after eight radial transects. After this point, measuring more transects did not significantly alter the SD.

Once an optimum number of transects per-VTS had been established, the optimization method was modified to find the minimum number of VTSs required per-SRCT dataset to generate mean increment width measurements representative of the entire specimen. The optimum number of transects were measured within the ROIs of 13 VTSs spread throughout the roots of the same five specimens (the maximum sample of VTSs that ensures independent sampling along the 630 µm longitudinal/z-axis of each dataset – 30 µm spacing providing one unsampled VTS in between each sampled VTS). VTSs were sampled inward from each endpoint of the z-axis, with the moving mean GLG width and SD calculated with each new VTS sampled. SD was regressed against the number of VTSs, and the optimum VTS number was determined as the number at-which the regression flattened ($-0.01 <$ change in SD/increase in VTS < 0.01).

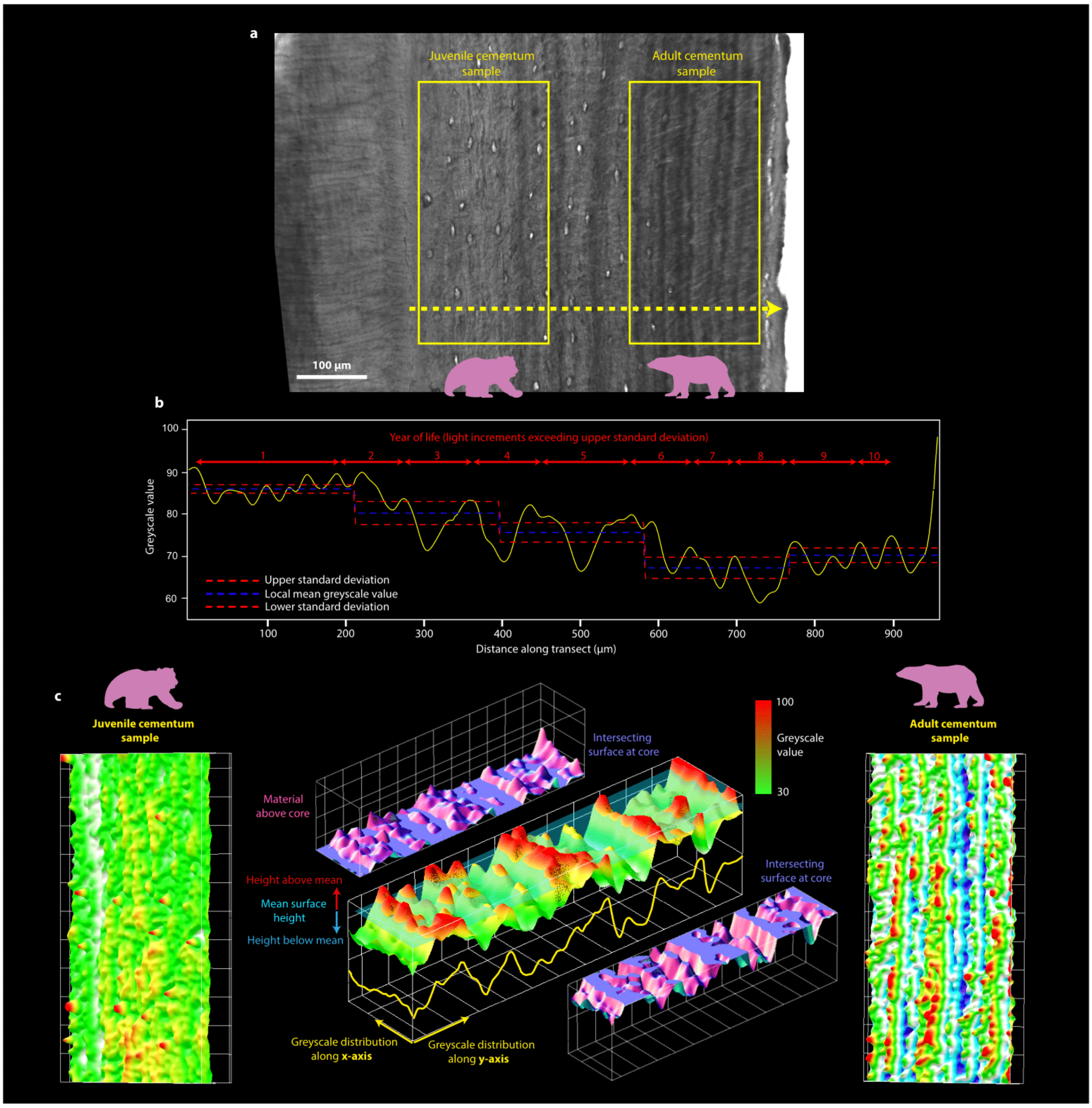

Fig. S1. Schematic description of cementum increment counting, width measurement, and texture analysis of juvenile and adult cementum. (**A**) Digital micrograph of *Ursus maritimus* cementum histological thin section. Yellow boxes represent subsamples of juvenile and adult cementum. Dashed yellow line represents 10-pixel wide transect shown in **B.** (**B**) Greyscale values (yellow line) measured along transect shown in **A**. Transects split into five equal portions to count and measure annual growth-layer-groups. Growth-layer-groups defined by consecutive light increments (peaks in greyscale) that exceed the upper standard deviation of the respective portion. **(c)** Comparison of juvenile and adult cementum data using 3D surface

profiling measures to characterize greyscale “texture” (spatial distribution). Four types of measures were used: 1) height measures: greyscale values relative to the mean value; 2) spatial measures: isotropy and directionality in greyscale along the *x*- versus *y*-axis; 3) hybrid measures: combined height and spatial measures; and 4) functional measures: greyscale distribution relative to intersects cut into the surface within its ‘core’ values. Silhouettes obtained from phylopic (http://phylopic.org/) under a Public Domain license.

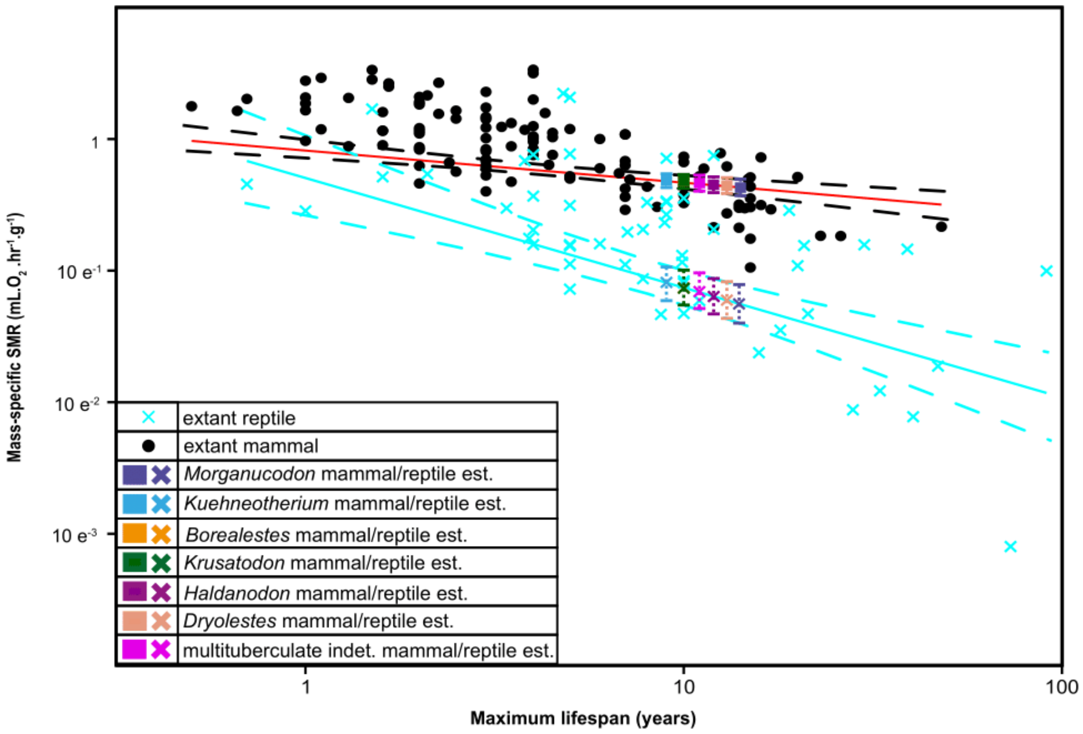

Fig. S2. Phylogenetically informed least-squares biplot between log-transformed maximum wild lifespan (years) and log-transformed mass-specific standard metabolic rate (msSMR; mL O_2 h^{-1} kg^{-1}) for extant mammals (n = 117) and reptiles (n = 55) (Data from Newham et al. (*4*)), and respective msSMR estimation for Jurassic mammaliaforms using each of these regressions.

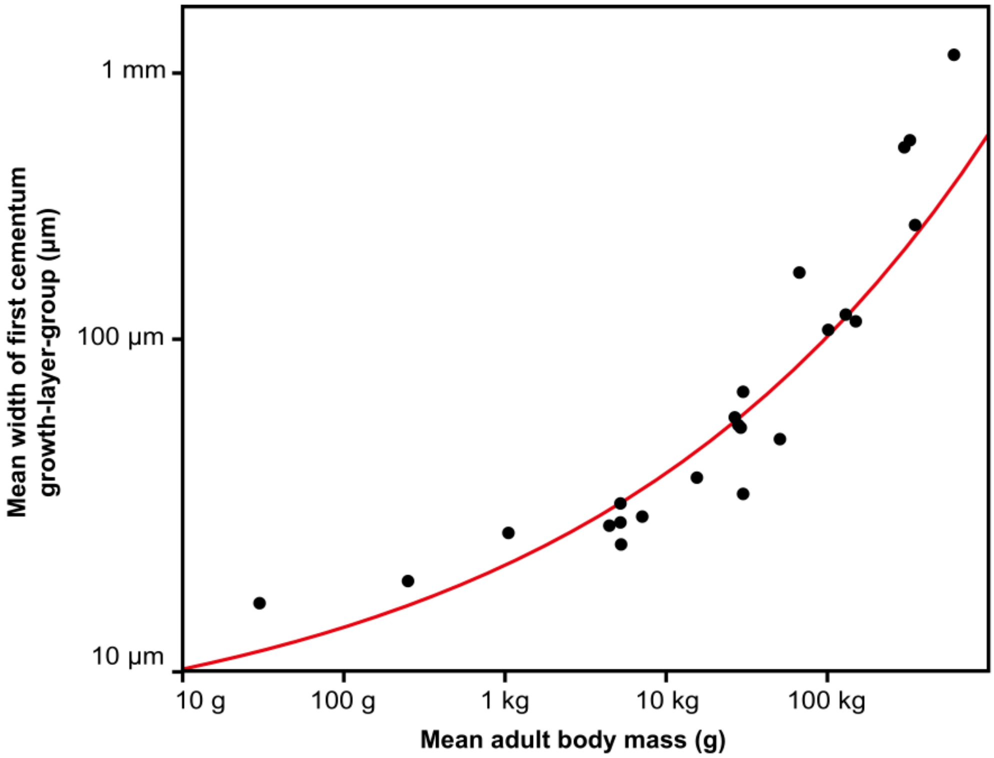

Fig. S3. Phylogenetically-informed exponential regression between mean adult body mass (g) and mean width of first cementum increment growth layer-group (µm) for 23 extant mammals. Source data are provided as a Source data file.

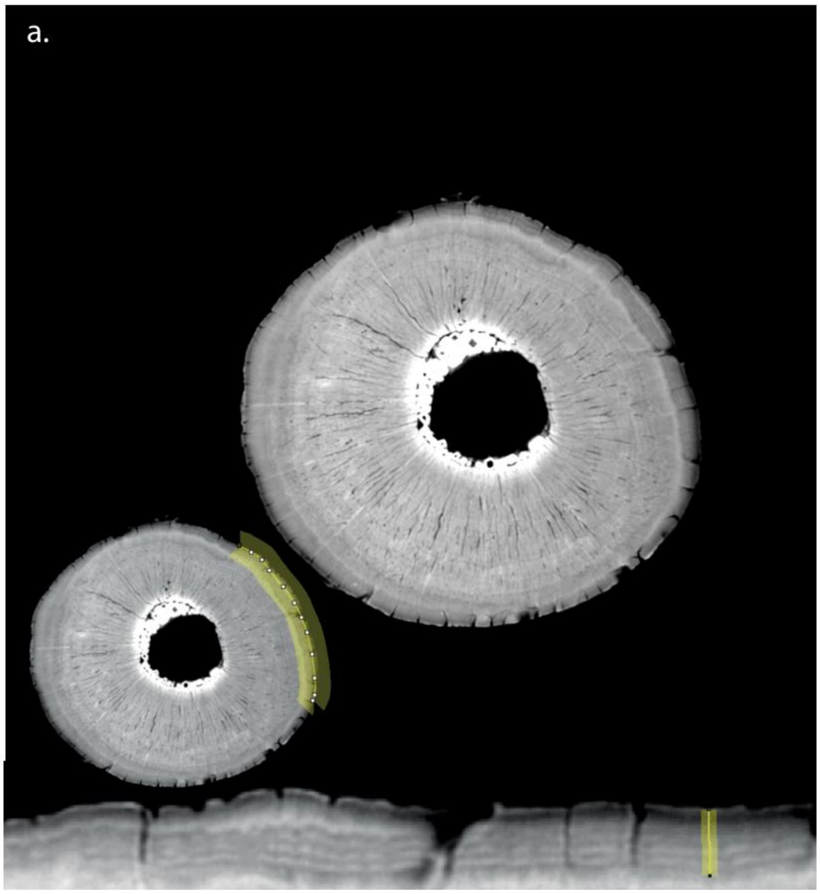
a.

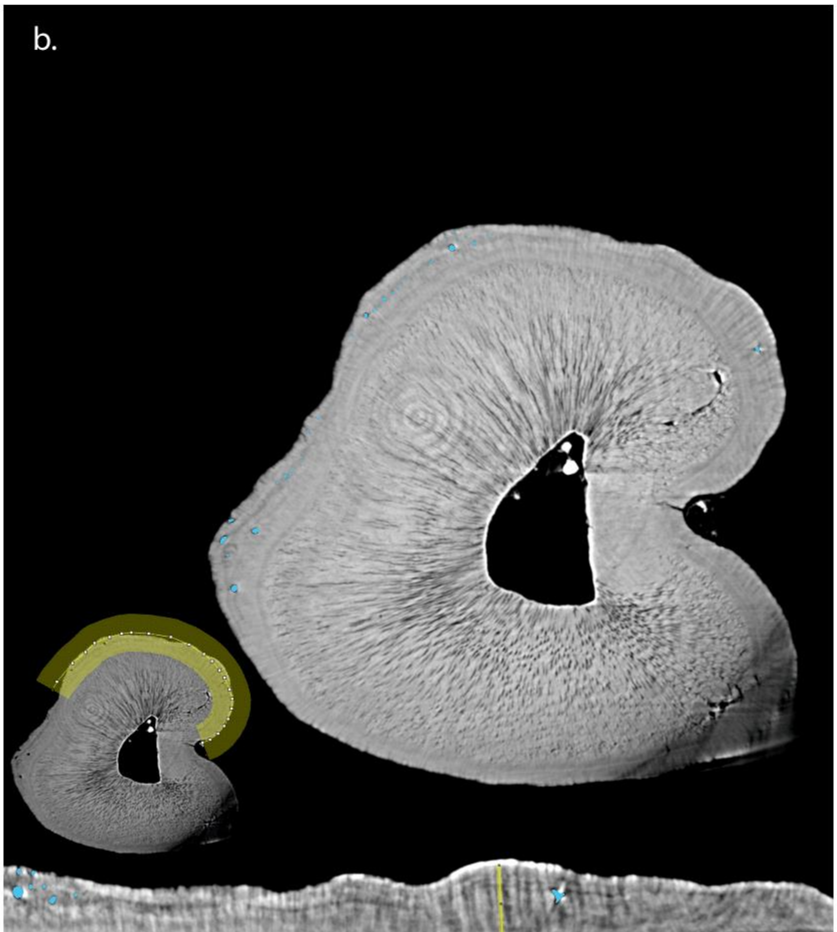
b.

Fig. S4. Comparison of cementum tissue types identified in SRCT data. (**A**) *Palaeoxonodon* specimen NHMUK PV M 46388 displaying only Acellular Extrinsic Fibre (AEFC) cementum, with no cellular voids and consistent incrementation. (**B**) *Haldanodon* specimen Gui Mam 3207 displaying a mixture of AEFC and Cellular Intrinsic Fibre (CIFC) cementum, with cellular voids highlighted in blue. CIFC isolated by clusters of cellular voids and disruption of AEFC incrementation.

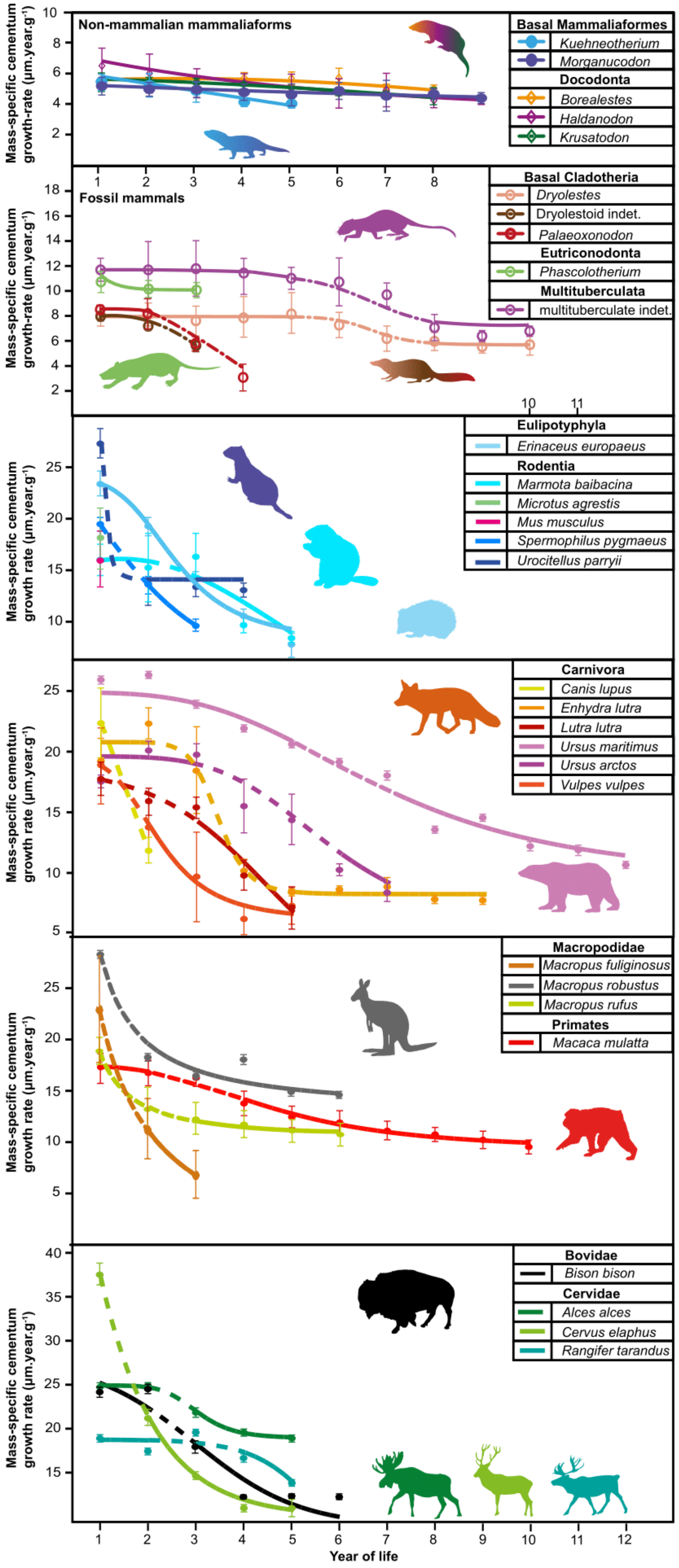

Non-mammalian mammaliaforms
Basal Mammaliaformes
Kuehneotherium
Morganucodon
Docodonta
Borealestes
Haldanodon
Krusatodon
Fossil mammals
Basal Cladotheria
Dryolestes
Dryolestoid indet.
Palaeoxonodon
Eutriconodonta
Phascolotherium
Multituberculata
multituberculate indet.
Eulipotyphyla
Erinaceus europaeus
Rodentia
Marmota baibacina
Microtus agrestis
Mus musculus
Spermophilus pygmaeus
Urocitellus parryii
Carnivora
Canis lupus
Enhydra lutra
Lutra lutra
Ursus maritimus
Ursus arctos
Vulpes vulpes
Macropodidae
Macropus fuliginosus
Macropus robustus
Macropus rufus
Primates
Macaca mulatta
Bovidae
Bison bison
Cervidae
Alces alces
Cervus elaphus
Rangifer tarandus
Mass-specific cementum growth-rate (µm.year.g⁻¹)
Mass-specific cementum growth rate (µm.year.g⁻¹)
Year of life

Fig. S5. Mass-specific cementum growth rates (msGRs) through life measured for fossil mammaliaform and full extant mammal sample. Data restricted to years-of-life represented by $\geq$ 3 specimens (for full lifespan estimates see Fig. 3). Dashed portions of extant plots represent the mean period for attainment of sexual maturity for the respective taxon. Bracketed vertical lines represent standard deviations for subsampled measurements (Materials and Methods). Symbols (see legends) represent mean msGRs for the respective taxon during the respective year of life. Lines for all taxa represent best-fitting non-linear models (Table 1). Dash-dotted portions of fossil crown mammal plots represent estimated periods for the attainment of sexual maturity.

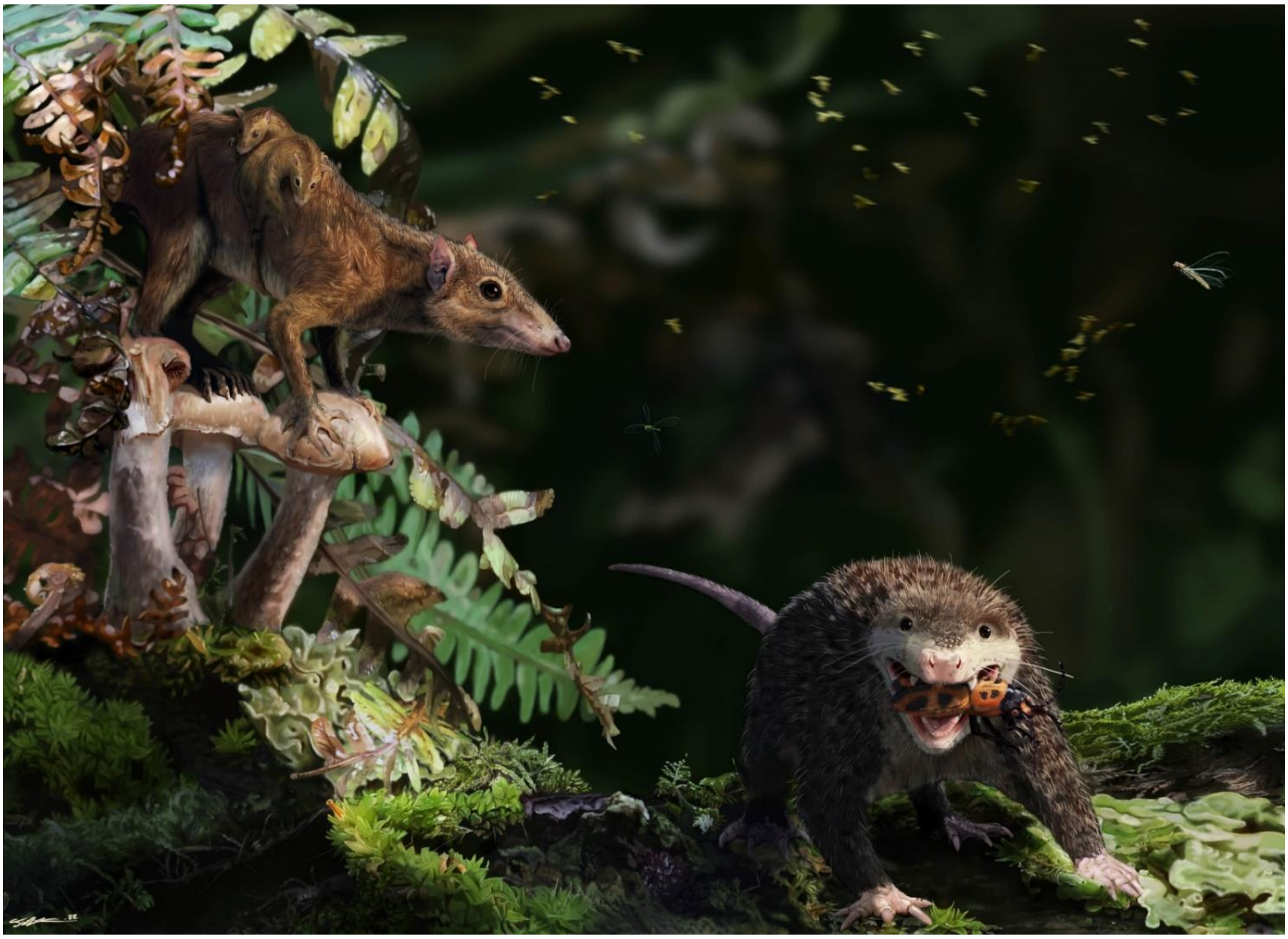

Fig. S6. Reconstruction of adult ($\geq$4 years old) *Dryolestes* (upper left) adult supporting juveniles, and *Haldanodon* (lower right) foraging in the Guimarota swamp. Artist, James Brown. Artwork commissioned by PGG, who holds copyright and permission to use for this publication.

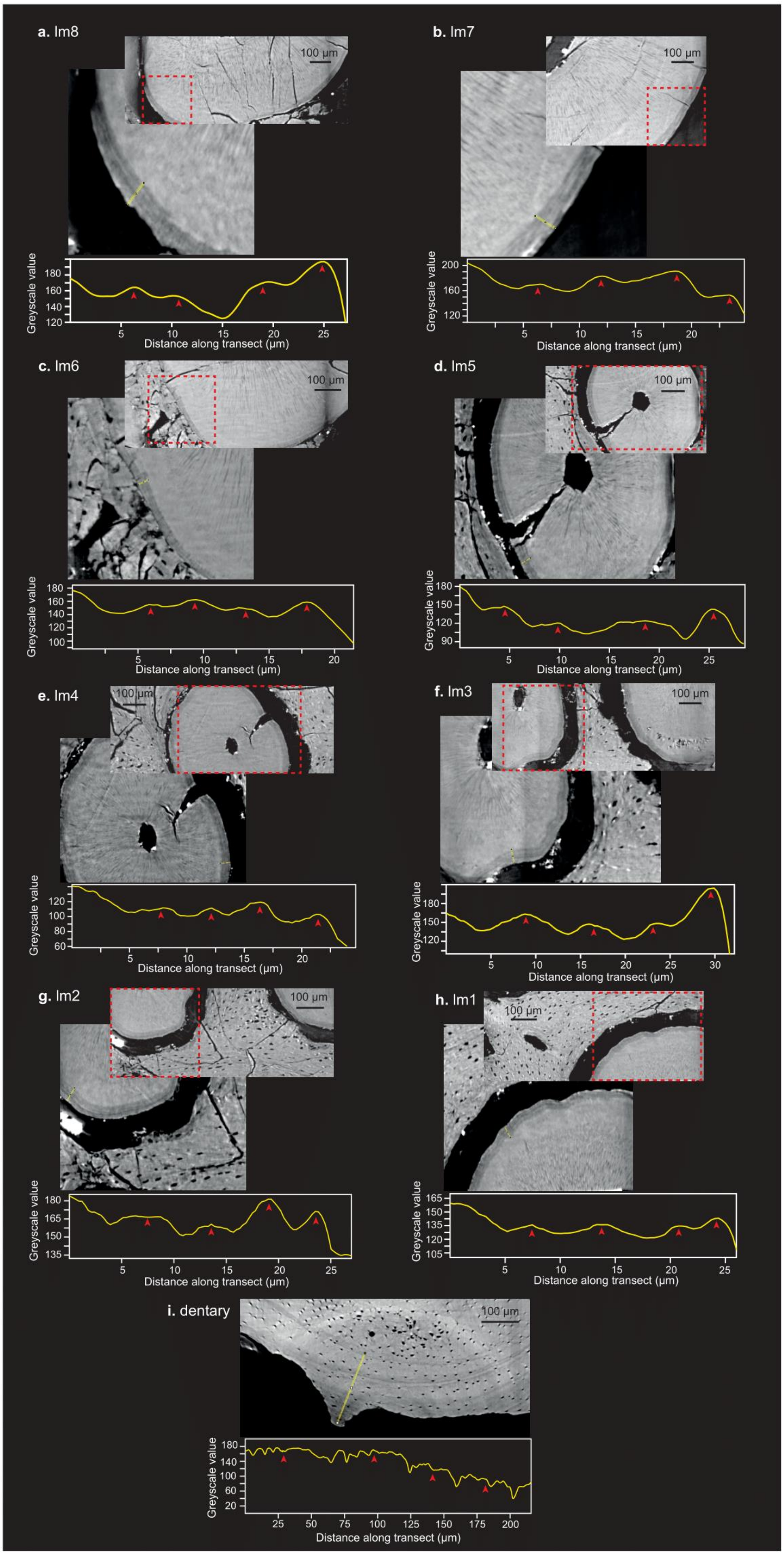

a. lm8
100 µm
Greyscale value
Distance along transect (µm)
b. lm7
100 µm
Greyscale value
Distance along transect (µm)
c. lm6
100 µm
Greyscale value
Distance along transect (µm)
d. lm5
100 µm
Greyscale value
Distance along transect (µm)
e. lm4
100 µm
Greyscale value
Distance along transect (µm)
f. lm3
100 µm
Greyscale value
Distance along transect (µm)
g. lm2
100 µm
Greyscale value
Distance along transect (µm)
h. lm1
100 µm
Greyscale value
Distance along transect (µm)
i. dentary
100 µm
Greyscale value
Distance along transect (µm)

Fig. S7. SRCT data and greyscale transects plotted across the cementum in teeth lm8 (**A**) to lm1 (**H**) for *Dryolestes* specimen Gui Mam 82/79. Transects denoted by yellow lines within detailed regions of interest marked using dashed red lines in the original SRCT slices. Counts of growth-layer-groups (four in each tooth type, denoted by red arrows) correspond to the counts of lines-of-arrested-growth (LAGs) found in the dentary of the same specimen (**i**), with the larger proportion of material extending after the fourth LAG suggesting that eruption of the molariform teeth occurred relatively late in the first year-of-life.

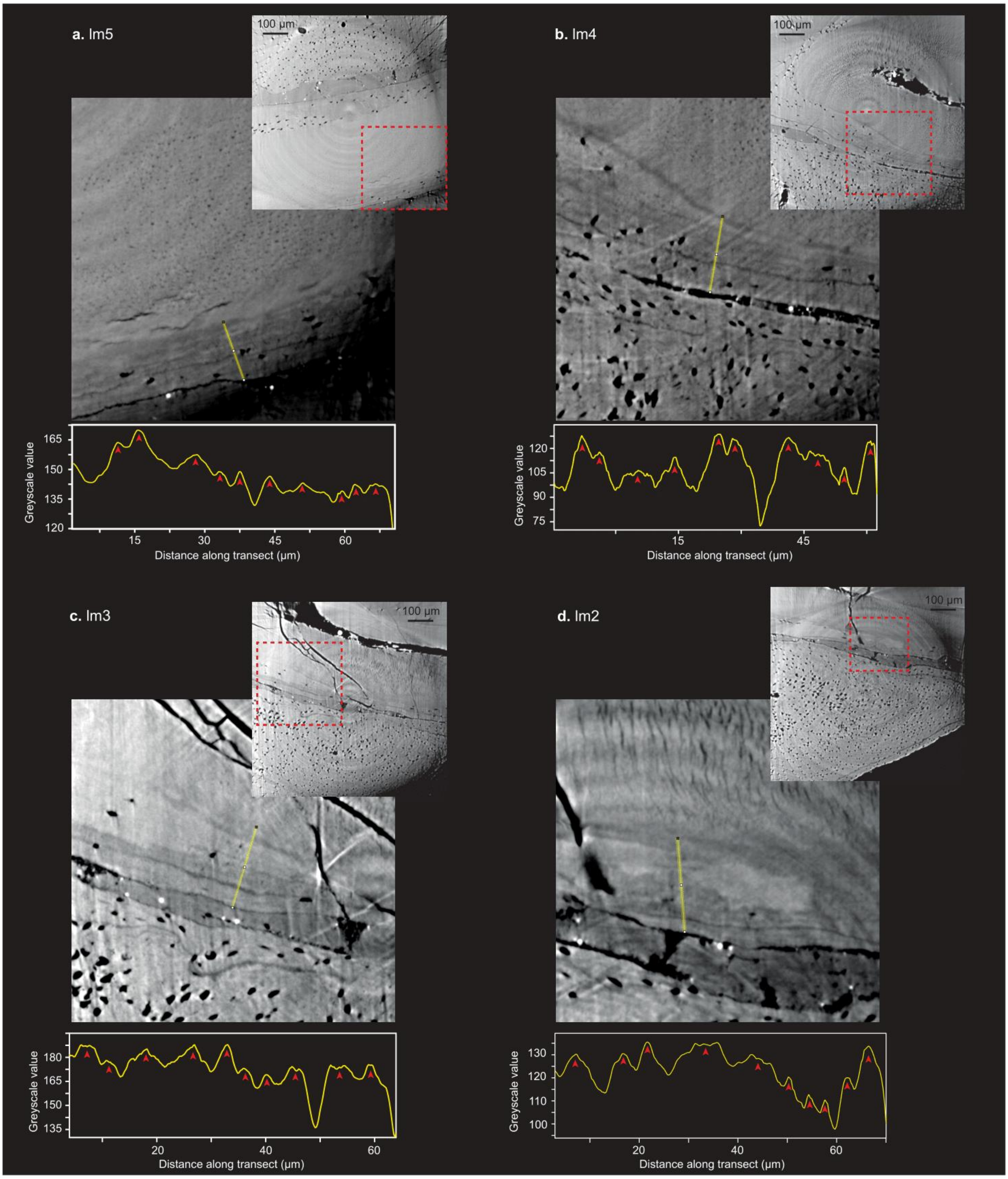
a. lm5
100 µm
Greyscale value
165
150
135
120
15
30
45
60
Distance along transect (µm)
b. lm4
100 µm
Greyscale value
120
105
90
75
15
45
Distance along transect (µm)
c. lm3
100 µm
Greyscale value
180
165
150
135
20
40
60
Distance along transect (µm)
d. lm2
100 µm
Greyscale value
130
120
110
100
20
40
60
Distance along transect (µm)

Fig. S8. SRCT data and greyscale transects plotted across the cementum in teeth lm5 (**A**) to lm2 (**D**) for *Haldanodon* specimen Gui Mam 3/80. Transects denoted by yellow lines within detailed regions of interest marked using dashed red lines in the original SRCT slices. Ten cementum growth-layer-groups were counted for each tooth type, denoted by red arrows.

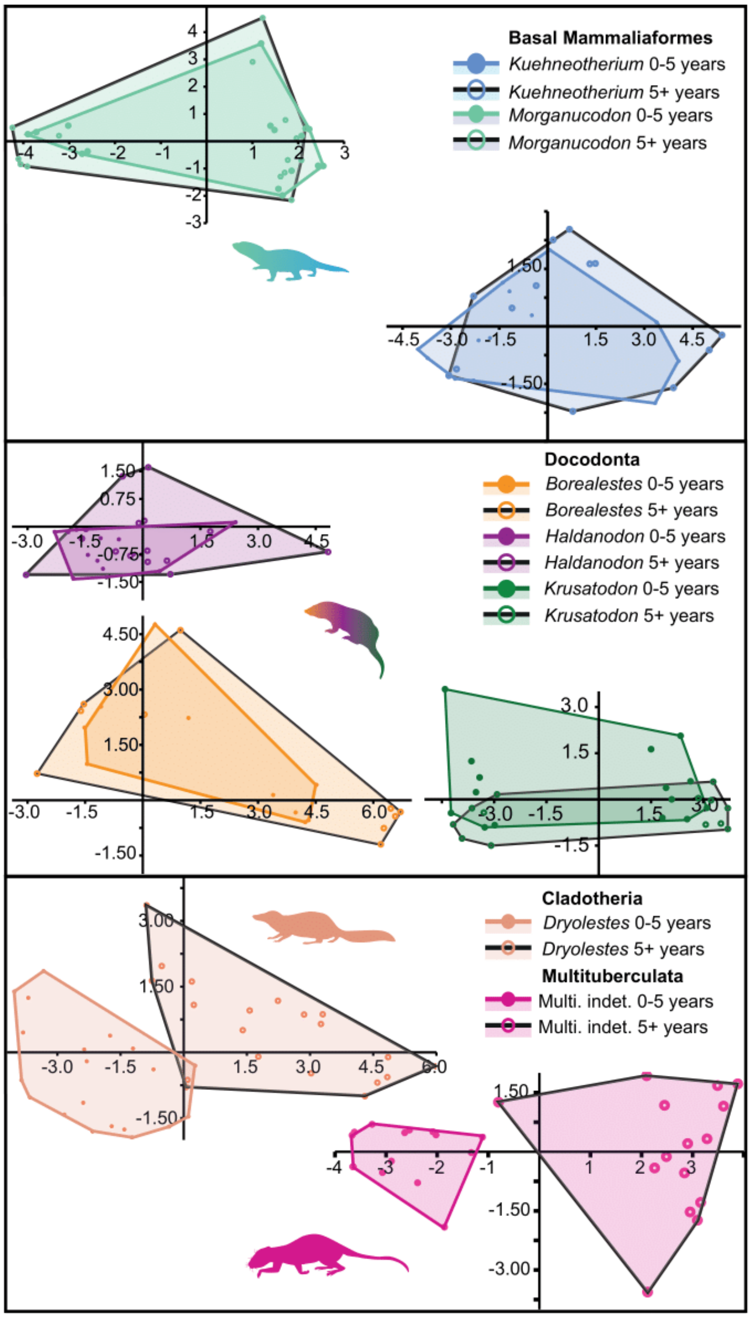

Fig. S9. Principal components plots comparing results of texture measures for 16-bit juvenile versus adult cementum texture for fossil mammaliaforms.

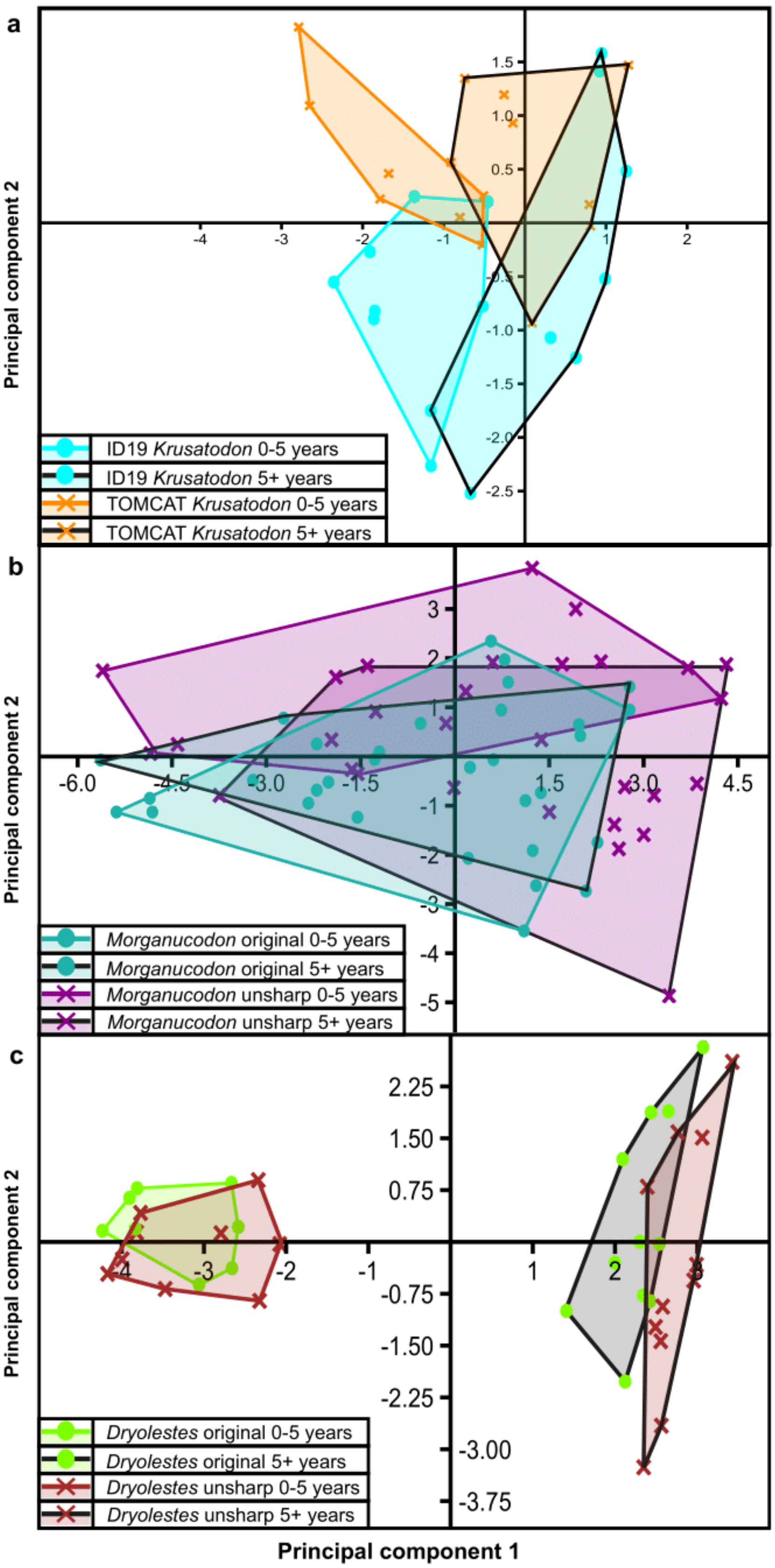

a
b
c
Principal component 2
Principal component 1
ID19 Krusatodon 0-5 years
ID19 Krusatodon 5+ years
TOMCAT Krusatodon 0-5 years
TOMCAT Krusatodon 5+ years
Morganucodon original 0-5 years
Morganucodon original 5+ years
Morganucodon unsharp 0-5 years
Morganucodon unsharp 5+ years
Dryolestes original 0-5 years
Dryolestes original 5+ years
Dryolestes unsharp 0-5 years
Dryolestes unsharp 5+ years

Fig. S10. (**A**) Principal Components plot comparing results of texture measures for cementum data generated at different synchrotron beamlines for the docodont *Krusatodon*. (**b-c**) Principal Components plots comparing results of texture measures for cementum data processed using an unsharp mask versus original data for *Morganucodon* (**B**) and *Dryolestes* (**C**).

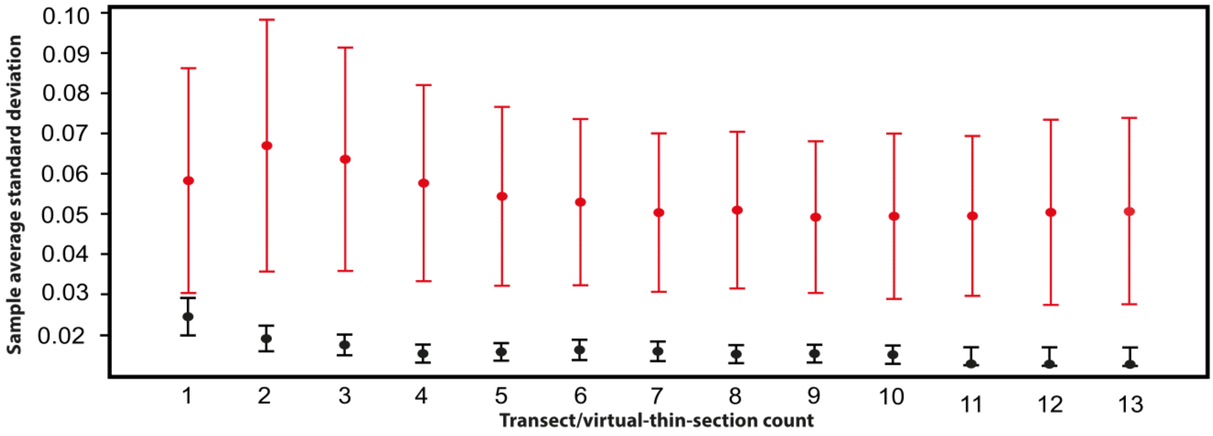

Fig. S11. Mean-whisker plot of average standard deviation in growth-layer-group width versus number of transects in a single VTS (red), and number of VTS studied per-dataset (black – 8 transects measured per-VTS) for entire subsample used for validation of cementum increment measuring methodology (Supplementary Note 5). Whiskers represent the upper and lower quartile range.

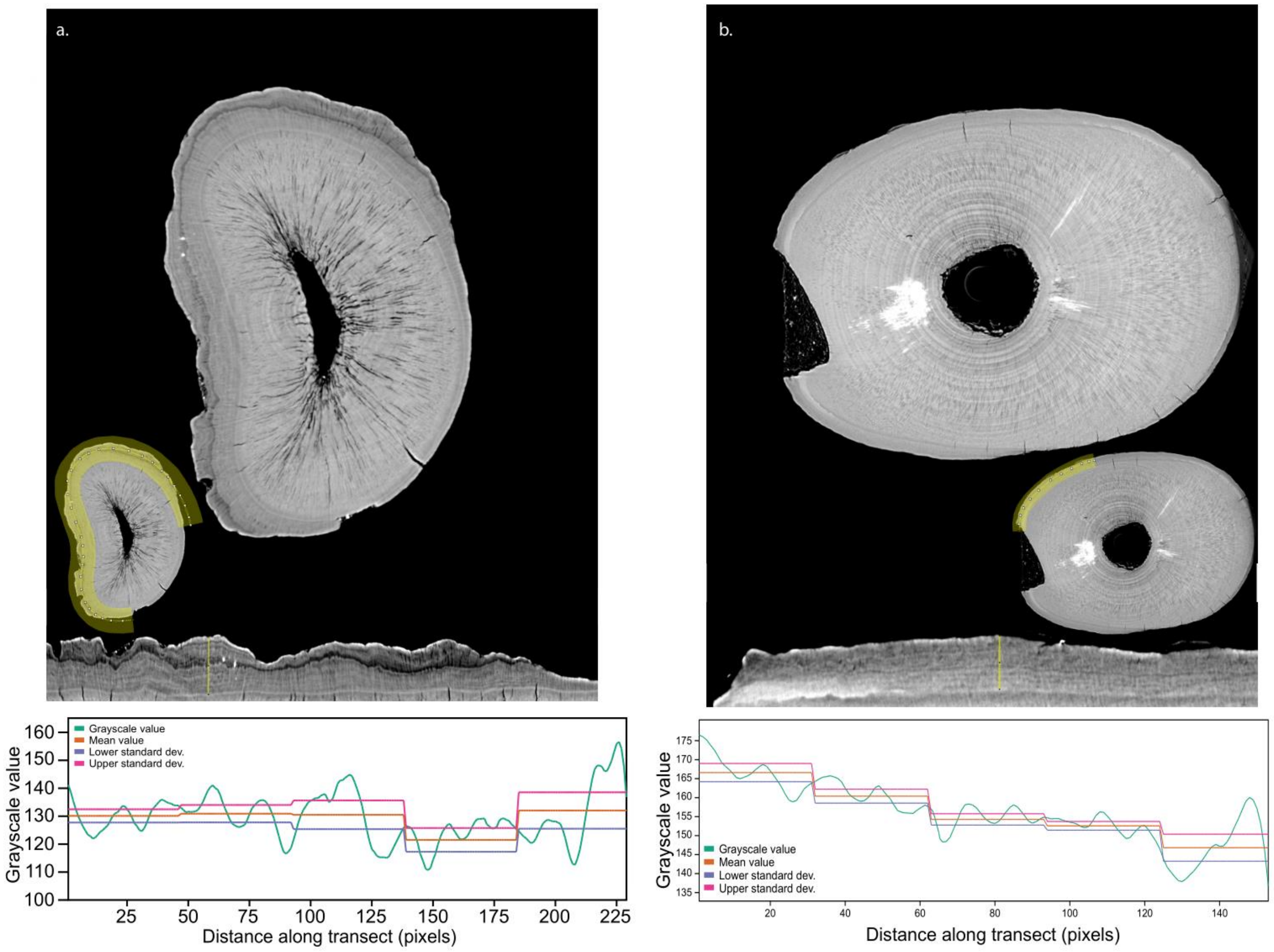
a.
b.
Grayscale value
Mean value
Lower standard dev.
Upper standard dev.
Grayscale value
Distance along transect (pixels)
Grayscale value
Mean value
Lower standard dev.
Upper standard dev.
Grayscale value
Distance along transect (pixels)

Fig. S12. Examples of category "a" specimens. (**A**) *Dryolestes* specimen Gui Mam 1191. (**B**) Multituberculate specimen Gui Mam 4207. Yellow bands in low resolution images represent cementum regions of interest straightened in lower image segments. Yellow transects in straightened segments represent radial transects through the cementum, measured in grayscale as represented by grayscale graphs below.

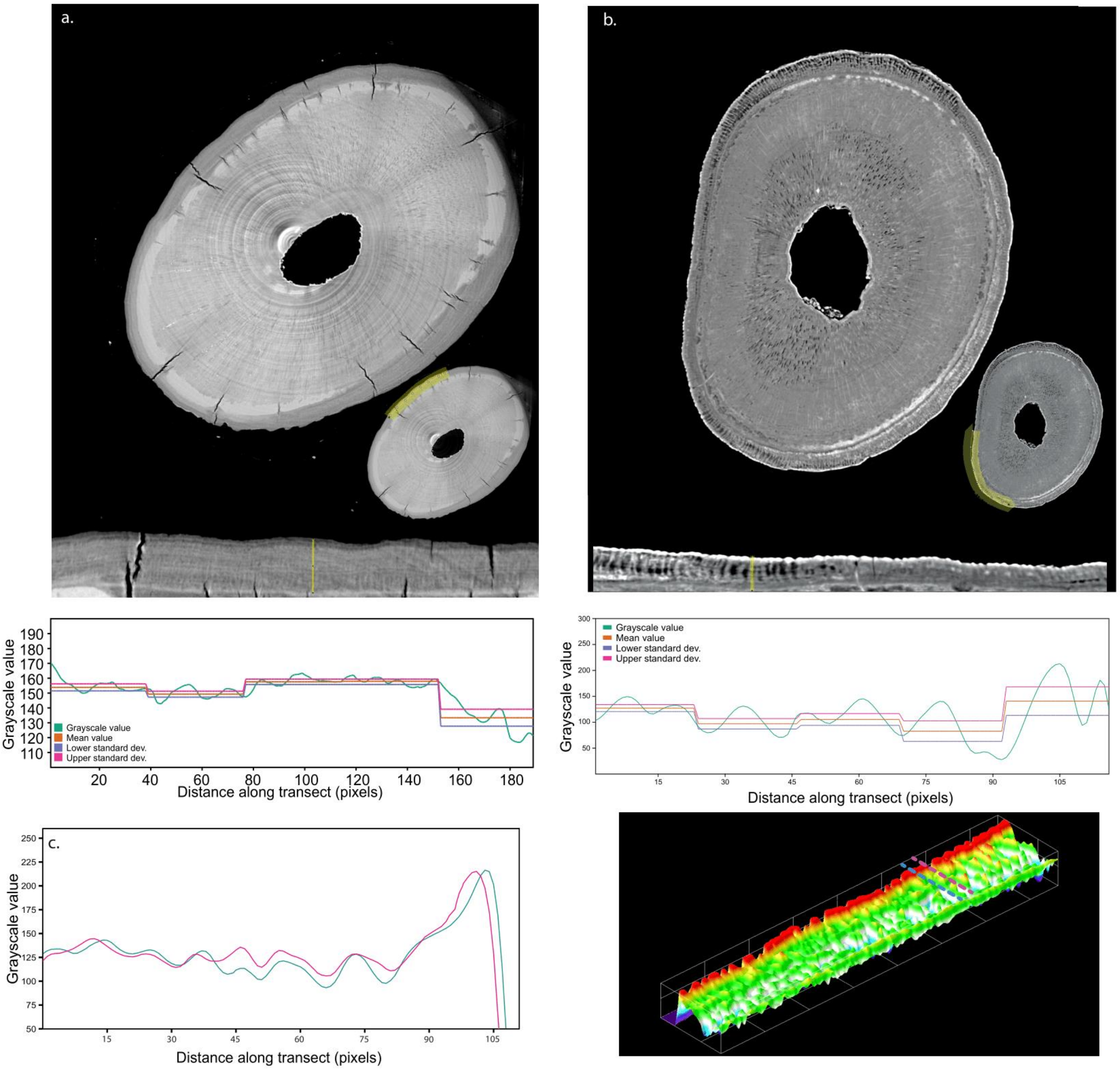
a.
b.
c.
Grayscale value
Mean value
Lower standard dev.
Upper standard dev.
Grayscale value
Distance along transect (pixels)
190
180
170
160
150
140
130
120
110
20
40
60
80
100
120
140
160
180
300
250
200
150
100
50
15
30
45
60
75
90
105
225
175
125
75

Fig. S13. Examples of category "b" specimens. (**A**) Multituberculate specimen Gui Mam 4209. (**B**) *Borealestes* specimen NHMUK PV M 100072. Yellow bands in low resolution images represent cementum regions of interest straightened in lower image segments. Bottom segment in (**C**); three-dimensional rendering of straightened section displaying radial cementum increment transects appended upon by circumferential bundles of persevered Sharpey's fibers. Highly correlative patterns in greyscale values across transects plotted in (**c**) across a Sharpey's fiber bundle (pink transect in **c**, position indicated by dashed pink line in 3D profile), and neighboring cementum region (blue transect in **c**, position indicated by dashed blue line in 3D profile) suggest that preserved Sharpey's fibers modify, but do not disturb, circum-annual radial cementum incrementation. Yellow transects in straightened segments represent radial transects through the cementum, measured in grayscale as represented by grayscale graphs below.

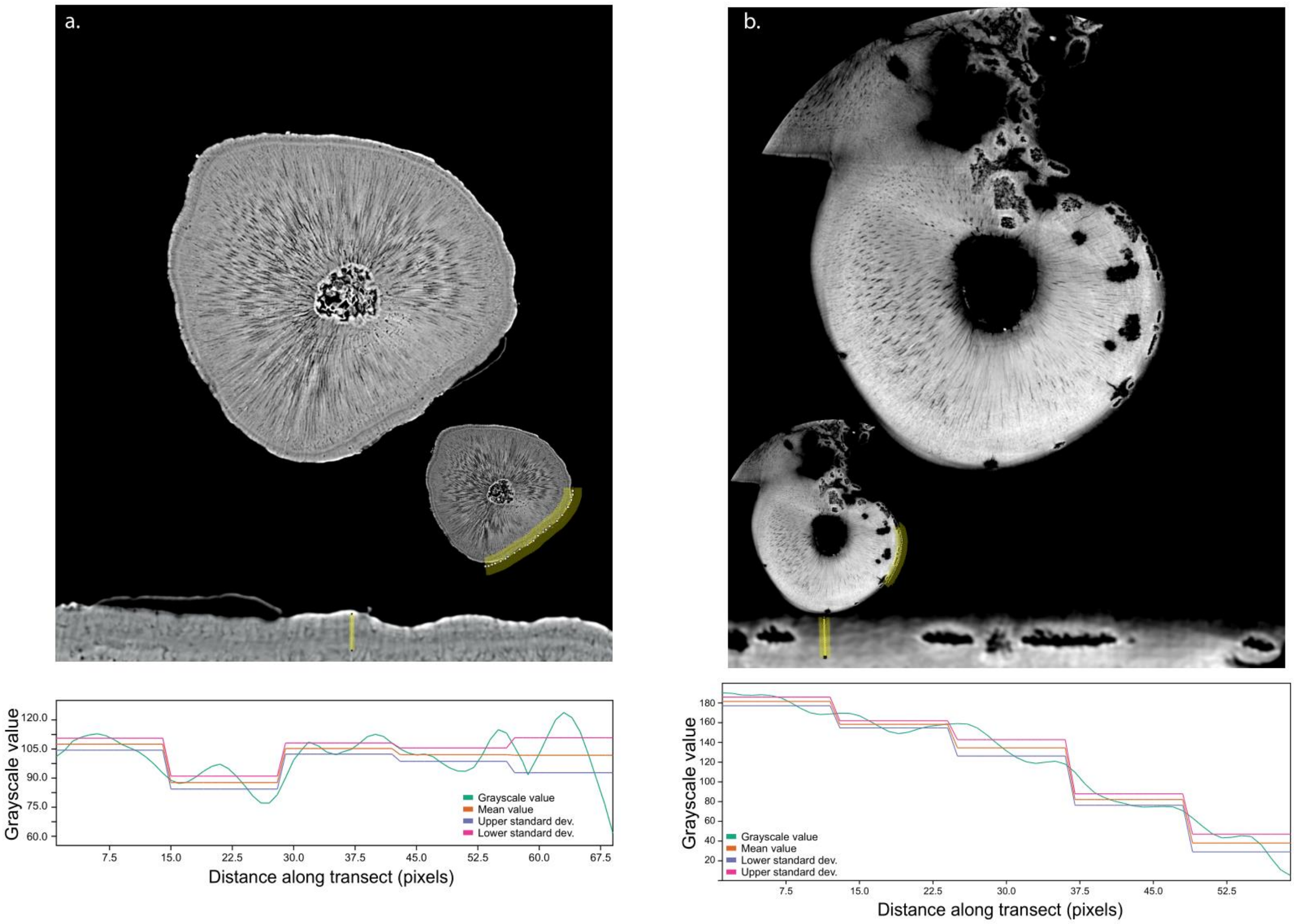
a.
b.
Grayscale value
120.0
105.0
90.0
75.0
60.0
7.5
15.0
22.5
30.0
37.5
45.0
52.5
60.0
67.5
Distance along transect (pixels)
Grayscale value
Mean value
Upper standard dev.
Lower standard dev.
Grayscale value
180
160
140
120
100
80
60
40
20
7.5
15.0
22.5
30.0
37.5
45.0
52.5
Distance along transect (pixels)
Grayscale value
Mean value
Lower standard dev.
Upper standard dev.

Fig. S14. Examples of category "c" specimens. (**A**) *Borelestes* specimen OXMNH J 79522, displaying common cellular voids indicative of cellular intrinsic fibred cementum and unclear incrementation. (**B**) Dryolestidan specimen OXMNH J 79445, displaying acellular intrinsic fibred cementum disturbed by taphonomic damage. Yellow bands in low resolution images represent cementum regions of interest straightened in lower image segments. Yellow transects in straightened segments represent radial transects through the cementum, measured in grayscale as represented by grayscale graphs below.

Taxon (superscript number refers to citation number - see References)	Sample size	Data type	Maximum estimated sample lifespan (years)	Mean known/estimated body mass (g)	Maximum known wild lifespan (years) (superscript number refers to citation number - see References)	Minimum age at sexual maturity (years)	Maximum age at sexual maturity (years)	Minimum age at cementum growth rate reduction	Maximum age at cementum growth rate reduction	First year msGR (µm.year. g-1)	Final year msGR (µm.year. g-1)	Best fitting cementum growth model				
												Type	Function	AIC	r^2	Min. annual slope
Morganucodon	11	SRCT	14	17.9	Na	Na	Na	Na	Na	5.22	4.42	Quadratic	0.003* year of life ^2 - 0.112* year of life +5.282	10.89	0.822	-0.324
Kuehneotherium	6	SRCT	9	23.8	Na	Na	Na	Na	Na	5.45	4.05	Quadratic	-0.005*year of life^2 – 0.364*year of life + 5.90	10.09	0.946	-0.70
Palaeoxonodon	3	SRCT	5	16.88	Na	Na	Na	Na	Na	8.35	4.22	von Bertanffy	5.823*exp(-2.196*year of life)+10.06	30.34	0.973	-1.99
Krusatodon	4	SRCT	10	45.81	Na	Na	Na	Na	Na	5.43	4.51	Gaussian	5.745*exp(-((year of life + 2.76)^2) / (2 * 224.1))	12.142	0.887	-0.506
Borealestes	5	SRCT	10	32.19	Na	Na	Na	Na	Na	5.40	5.04	Gaussian	5.68 * exp(-((year of life – 2.037)^2) / (2 * 125.344))	12.073	0.879	-0.341
Dryolestes	13	SRCT	13	130	Na	Na	Na	5	7	7.99	5.69	Hill's sigmoidal	7.934 / (1 + (year of life / 6.424)^1)	16.314	0.97	-1.508
Haldanodon	9	SRCT	12	64	Na	Na	Na	Na	Na	6.47	4.26	Exponential	3.43*exp(-0.164*year of life) + 3.51	11.03	0.949	-0.69
Phascolotherium	3	SRCT	3*	70.75	Na	Na	Na	2	3	10.820	9.944	Exponential	5.823*exp(-2.196*year of life) + 10.06	Na	1	-0.716
Dryolestida indet.	5	SRCT	6*	25.01	Na	Na	Na	3	4	7.94	5.61	Quadratic	-0.402*year of life^2 +	Na	1	-1.570

													0.444*year of life + 7.898			
multituberculate indet.	9	SRCT	11	45	Na	Na	Na	5	7	11.67	8.41	Hill's sigmoidal	11.688 / (1+(year of life / 7.037)^5.328)	16.14	0.992	-2.035
Erinaceus europaeus	5	SRCT	8	750	8[96]	0.9	1	1	3	23.647	7.990	Hill's sigmoidal	23.996 / (1 + (year of life / 2.558)^3.841)	27.674	0.973	-5.750
Macaca mulatta	10	SRCT	10	8235	36[97]	2	4	1	4	15.010	8.850	Hill's sigmoidal	15.88 / (1 + (year of life / 4.564)^3.13)	16	1	-1.400
Ursus maritimus[30]	6	histology	15	300000	32[98]	3	7	3	7	26.307	10.810	Hill's sigmoidal	25.984 / (1 + (year of life / 6.853)^3.356)	31.037	0.961	-2.160
Macropus robustus[91]	9	histology	8	30000	22[99]	1	2	1	2	25.735	11.240	Hill's sigmoidal	1559.5 / (1 + (year of life / 0.021)^1.2284)	21.195	0.932	-7.800
Macropus rufus[91]	5	histology	7	50750	30[99]	1	3	1	3	19.858	10.200	Hill’s sigmoidal	5.45.09 / (1 + (year of life / 0.0750)^1.5257)	18.23	0.996	-6.81
Macropus fuliginosus[91]	3	histology	7	28000	20[100]	1	2	1	?	27.565	15.449	Power	20.884* year of life^-0.791 + 6.70	Na	1	-8.808
Alces alces[92]	3	histology	5	386000	22[101]	2	3	2	3	25.463	19.352	Hill's sigmoidal	25.464 / (1 + (year of life/2.999)^7.976)	18.732	1	-3.069
Cervus elaphus[93]	3	histology	5	200000	21[102]	1	2.5	1	3	38.170	11.487	exponential	67.947*exp(-0.888*year of life) + 10.23	30.73	0.999	-16.371
Rangifer trarandus[93]	3	histology	15	101250	15[102]	2	4	2	4	19.458	14.310	logistic	19.199 / (1+ 0.0003 *exp(+1.414*year of life))	33.5	0.832	-4.880
Lutra lutra[93]	3	histology	5	7100	16[100]	1	2	1	4	18.193	7.479	gaussian	17.945 * exp(-((year of life - 1.115)^2) / (2 *8.419))	32.91	0.964	-5.542

Canis lupus[22]	3	histology	2	26625	13[103]	1	2	1	2	22.750	12.258	linear	-10.493*year of life + 33.244	NA	NA	-10.493
Bison bison[22]	3	histology	6	610000	25[100]	2	3	1	4	20.899	4.599	logistic	25.979 / (1 + 0.064 * exp(+0.918 * year of life))	47.17	0.911	-9.015
Spermophilus pygmaeus[22]	4	histology	3	250	7.1[104]	1	2	1	2	19.705	9.845	quadratic	1.009*year of life^2 - 8.964*year of life + 27.662	NA	1	-5.939
Marmota baibacina[22]	3	histology	5	5250	14[105]	2	3	2	4	16.216	10.119	Hill's sigmoidal	16.064 / (1 + (year of life / 3.486)^109.94)	40.983	0.985	-6.681
Ursus arctos[22]	3	histology	7	130000	30[106]	3.5	6	2	6	19.004	8.746	Hill's sigmoidal	19.986 / (1 + (year of life / 5.371)^4.747)	32.595	0.964	-4.239
Urocitellus parryii[22]	3	histology	5	1500	10[107]	1	2	1	2	27.545	16.588	Hill's sigmoidal	29.234 / (1 + (year of life / 1.0615)^34.494)	43.369	0.952	-13.646
Vulpes vulpes[22]	5	histology	5	4440	12[108]	<1	1	1	2	19.210	7.659	Exponential	24.495*exp(-0.525*year of life) + 4.953	33.31	0.969	-5.058
Microtus agrestis	4	histology	1	30	1[109]	<1	<1	Na	Na	19.026	19.026	Na	Na	Na	Na	Na
Mus musculus	5	histology	1	20.5	1[110]	<1	<1	Na	Na	16.020	16.020	Na	Na	Na	Na	Na
Enhydra lutra[29]	32	histology	9	24500	23[111]	2	5	2	4	19.620	8.043	Hill's sigmoidal	21.153 / (1+(year of life / 3.428)^11.213)	23.892	0.978	-8.267
Ovis aries[94]	27	histology	Na	150000	22.8[112]	1.8	2.5	Na	Na	18.209	Na	Na	Na	Na	Na	Na
Vulpes lagopus[95]	21	histology	Na	5200	16.3[113]	0.9	1	Na	Na	18.904	Na	Na	Na	Na	Na	Na
Raphicerus campestris[92]	5	histology	Na	11500	9.3[97]	1	1.5	Na	Na	29.54	Na	Na	Na	Na	Na	Na

Supplement Table 1. Cementum growth and life history variables for full sample of fossil and extant mammals. Minimum annual slope value indicates greatest slope between two consecutive years-of-growth (due to negative growth-rate models). * indicates maximum lifespan found, but not used to estimate maximum lifespan for the respective species due to growth patterns indicating insufficient sampling of adult individuals.

Supplementary Table 2. List of extant taxa used in cementum texture analysis.

**Taxon (superscript number refers to citation number - see References)**
Alces alces[93]
Cervus elaphus[93]
Erinaceus europaeus
Lutra lutra[29]
Macaca mulatta
Macropus robustus[91]
Rangifer tarandus[93]
Ursus maritimus[30]

Supplementary Table 3. Descriptions for surface profiling metrics employed to study cementum texture, minimum *F* value and maximum *p* value for the respective metric when values were compared between juvenile and adult data for extant cementum samples (Supplementary Table 2).

**Measure**	**Category**	**Description**	**Minimum *F* value**	**maximum *P* value**	**Taxon**
Absolute average height of surface	Height	Average greyscale value of sample.	8.51	0.015	*Alces alces*
Relative average height of surface	Height	Average difference between greyscale values above average value of sample.	10.61	0.008	*Lutra lutra*
Root mean square height of surface	Height	Root mean square (RMS) greyscale value.	5.56	0.040	*Lutra lutra*
Maximum peak height of surface	Height	Value of maximum greyscale peak above average value of sample.	5.29	0.039	*Alces alces*

Average maximum peak height	Height	Average value of 10 highest greyscale peaks above average value of sample.	6.80	0.030	*Alces alces*
Peak volume	Height	Average volume of peaks above average value of sample.	0.37	0.561	*Alces alces*
Minimum valley depth	Height	Value of lowest greyscale trough found below the average greyscale value of sample.	5.12	0.036	*Lutra lutra*
Average minimum valley depth	Height	Average value of 10 lowest greyscale troughs found below the average greyscale value of sample.	6.48	0.040	*Lutra lutra*
Average height	Height	Average height of greyscale peaks.	8.51	0.024	*Rangifer tarandus*
Ten-point average height	Height	10-point moving average value of greyscale peaks.	8.51	0.024	*Rangifer tarandus*
Maximum skew	Hybrid	Degree of symmetry of the surface heights about the mean plane.	0.37	0.578	*Lutra lutra*
Summit density	Hybrid	Number of greyscale peaks per unit area.	10.61	0.013	*Rangifer tarandus*
Maximum kurtosis	Hybrid	Prominence of inordinately high peaks and/or deep valleys.	0.01	0.924	*Lutra lutra*
Root mean square gradient of surface	Hybrid	Root mean square (RMS) of greyscale slopes, evaluated over all directions.	0.000	0.970	*Alces alces*

Developed interfacial area ratio	Hybrid	Percentage of additional surface area contributed by the texture as compared to an ideal plane the size of the measurement's region.	0.06	0.577	*Alces alces*
Core roughness depth	Functional	Proportion of dataset occupied by the core.	8.35	0.027	*Alces alces*
Average peak height above core	Functional	Average difference in greyscale between peaks above the maximum core greyscale value versus the maximum core greyscale value.	8.250	0.004	*Lutra lutra*
Average valley depth below core	Functional	average difference in greyscale between toughs below the minimum core greyscale value, and the minimum core greyscale value.	6.740	0.004	*Lutra lutra*
Surface bearing area ratio	Functional	Proportion of the surface that consists of peaks above the core greyscale value.	4.690	0.002	*Rangifer tarandus*
Autocorrelation length	Spatial	Horizontal distance of the autocorrelation function (ACF) that has the fastest decay to the value 0.2. Large value: surface dominated by low frequencies; low value: surface dominated by high frequencies.	0.000	0.957	*Alces alces*

Texture aspect ratio	Spatial	Ratio between the distance with the fastest to the distance with the slowest decay of the autocorrelation function (ACF).	0.000	0.97	*Alces alces*

Supplementary Table 4. List of all *Hirmeriella* specimens analyzed.

Specimen	*Taxon*	Estimated lifespan (years)	Preservational category
NHMUK PV M 104136	*Morganucodon*	2	c
NHMUK PV M 92528	*Morganucodon*	3	b
NHMUK PV M 95809	*Morganucodon*	3	c
NHMUK PV M 96490	*Morganucodon*	3	c
NHMUK PV M 104131	*Morganucodon*	4	b
NHMUK PV M 104134	*Morganucodon*	4	b
NHMUK PV M 96380	*Morganucodon*	4	b
NHMUK PV M 96396	*Morganucodon*	4	b
NHMUK PV M 104130	*Morganucodon*	5	c
NHMUK PV M 96441	*Morganucodon*	5	c
NHMUK PV M 104128	*Morganucodon*	5	a
NHMUK PV M 96413	*Morganucodon*	5	a
NHMUK PV M 96075	*Morganucodon*	8	b
NHMUK PV M 104129	*Morganucodon*	9	b
NHMUK PV M 104138	*Morganucodon*	10	a
NHMUK PV M 104127	*Morganucodon*	14	a
NHMUK PV M 21081	*Kuehneotherium*	2	c

NHMUK PV M 20990	*Kuehneotherium*	3	a
UMZC Sy 121	*Kuehneotherium*	4	b
NHMUK PV M 27443	*Kuehneotherium*	4	b
NHMUK PV M 21080	*Kuehneotherium*	5	b
NHMUK PV M 27550	*Kuehneotherium*	4	c
NHMUK PV M 27529	*Kuehneotherium*	5	c
NHMUK PV M 27537	*Kuehneotherium*	5	b
NHMUK PV M 20982	*Kuehneotherium*	5	a
NHMUK PV M 21069	*Kuehneotherium*	6	c
NHMUK PV M 27538	*Kuehneotherium*	6	c
NHMUK PV M 27545	*Kuehneotherium*	6	c
UMZC Sy 106	*Kuehneotherium*	8	c
NHMUK PV M 27653	*Kuehneotherium*	9	c

Supplementary Table 5. List of all Forest Marble specimens analyzed.

Specimen	Taxon	Estimated lifespan (years)	Preservational category
NHMUK PV M 46728	*Borealestes*	8	b
NHMUK PV M 46809	*Borealestes*	11	a
NHMUK PV M 46847	*Borealestes*	4	c
OXMNH J 79522	*Borealestes*	6	c
NHMUK PV M 36541	*Borealestes*	9	a
NHMUK PV M 100072	*Borealestes*	8	b
NHMUK PV M 46577	*Borealestes*	5	b
NHMUK PV M 46684	Dryolestidae indet.	6	b
OXMNH J 79445	Dryolestidae indet.	3	c

NHMUK PV M 100063	Dryolestidae indet.	3	a
NHMUK PV M 46045	Dryolestidae indet.	6	b
NHMUK PV M 46786	Dryolestidae indet.	2	b
NHMUK PV M 105719	Dryolestidae indet.	4	b
NHMUK PV M 46442	*Krusatodon*	10	b
NHMUK PV M 46531	*Krusatodon*	7	b
NHMUK PV M 36505	*Krusatodon*	9	a
NHMUK PV M 100062	*Krusatodon*	9	c
OXMNH J 79454	*Krusatodon*	3	c
OXMNH J 79523	*Krusatodon*	2	c
NHMUK PV M 46333	*Krusatodon*	1	b
NHMUK PV M 46197	*Palaeoxonodon*	4	b
NHMUK PV M 46213	*Palaeoxonodon*	4	b
NHMUK PV M 46388	*Palaeoxonodon*	5	a
NHMUK PV M 46429	*Palaeoxonodon*	6	c
NHMUK PV M 46702	*Palaeoxonodon*	3	c
NHMUK PV M 100065	*Phascolotherium*	3	a
NHMUK PV M 100092	*Phascolotherium*	3	b
NHMUK PV M 100060	*Phascolotherium*	5	b

Supplementary Table 6. List of all Guimarota specimens analyzed.

Specimen	Taxon	Estimated lifespan (years)	Preservational category
Gui Mam 1122	*Dryolestes*	4	c
Gui Mam 1137	*Dryolestes*	11	b
Gui Mam 1142	*Dryolestes*	5	c
Gui Mam 1151	*Dryolestes*	3	b
Gui Mam 1154	*Dryolestes*	2	b
Gui Mam 1164	*Dryolestes*	4	b
Gui Mam 1172	*Dryolestes*	5	c
Gui Mam 1173	*Dryolestes*	8	b
Gui Mam 1180	*Dryolestes*	4	b
Gui Mam 1183	*Dryolestes*	4	b
Gui Mam 1184	*Dryolestes*	4	b
Gui Mam 1185	*Dryolestes*	4	b
Gui Mam 1190	*Dryolestes*	10	a
Gui Mam 1191	*Dryolestes*	13	a
Gui Mam 1202	*Dryolestes*	6	b
Gui Mam 1212	*Dryolestes*	3	c
Gui Mam 1216	*Dryolestes*	3	a
Gui Mam 3139	*Haldanodon*	4	a
Gui Mam 3171	*Haldanodon*	6	b
Gui Mam 3207	*Haldanodon*	11	b
Gui Mam 3217	*Haldanodon*	6	b
Gui Mam 3238	*Haldanodon*	7	c
Gui Mam 3270	*Haldanodon*	4	b

Gui Mam 3282	*Haldanodon*	6	b
Gui Mam 3283	*Haldanodon*	4	b
Gui Mam 3284	*Haldanodon*	8	c
Gui Mam 3285	*Haldanodon*	9	b
Gui Mam 3286	*Haldanodon*	4	c
Gui Mam 3287	*Haldanodon*	9	b
Gui Mam 3288	*Haldanodon*	4	c
Gui Mam 4201	multituberculate	8	b
Gui Mam 4202	multituberculate	7	b
Gui Mam 4203	multituberculate	4	c
Gui Mam 4204	multituberculate	5	c
Gui Mam 4205	multituberculate	6	b
Gui Mam 4206	multituberculate	4	b
Gui Mam 4207	multituberculate	11	a
Gui Mam 4208	multituberculate	5	b
Gui Mam 4209	multituberculate	11	b
Gui Mam 4210	multituberculate	6	b
Gui Mam 4211	multituberculate	11	a

Supplementary Table 7. Summary of scanned and studied specimens for each synchrotron experiment. ESRF; European Synchrotron Radiation Facility, SLS; Swiss Light Source. Studied/useable sample size indicates the number of scanned specimens that comprised cementum of sufficient preservational quality for study.

Experiment	Dates	Beamline	Taxa studied	Sample size	Studied/usable subsample size
ES 152	18/04/2014 - 22/04/2014	ID19 (ESRF)	*Morganucodon*	71	17
			Kuehneotherium	2	0
			Erinaceus europaeus	5	5
20141278	13/04/15 - 16/04/15	TOMCAT (SLS)	*Morganucodon*	12	0
			Kuehneotherium	117	16
20151391	04/03/16 - 07/03/16	TOMCAT (SLS)	*Macaca mulatta*	10	10
20160404	18/11/16 - 21/11/16	TOMCAT (SLS)	Mammalia indet.	21	0
			amphilestid	4	0
			amphitheriid	1	0
			Amphitherium	2	0
			Borealestes	13	7
			Cyrtlatherium	3	0
			docodontan	12	0
			dryolestidan	4	4
			Eleutherodon	6	0
			Gobiconodon	1	0
			gobiconodontid	2	0
			haramiyidan	1	0
			Kermackodon	1	0
			Kirtlingtonia	1	0
			Krusatodon	7	3
			multituberculate	4	0
			Palaeoxonodon	2	0
			Phascolotherium	9	3
			Shuotherium	4	0
			Simpsonodon	8	0
			Wareolestes	1	0
ES-502	01/02/2017 - 05/02/2017	ID19 (ESRF)	"Symmetrodonta"	1	0
			Mammalia indet.	18	0
			amphilestid	2	0
			Borealestes	4	0

			docodontan	4	0
			dryolestidan	2	2
			eutriconodontan	1	0
			Gobiconodon	1	0
			haramiyidan	1	0
			Kennetherium	4	0
			Krusatodon	4	4
			morganucodontid	1	0
			Palaeoxonodon	23	5
ES-583	14/02/18 - 17/02/18	ID19 (ESRF)	Mammalia indet.	2	0
			Dryolestes	26	0
			Haldanodon	29	0
			Krebsotherium	17	0
			kuehneodon	2	0
			Paulchoffatia	1	0
20180876	13/09/18 - 16/09/18	TOMCAT (SLS)	Mammalia indet.	42	0
			docodontan	2	0
			Dryolestes	26	17
			Haldanodon	29	13
			multituberculate	16	11

Supplementary Table 8. Standard deviation of growth-layer-group measurements with increasing transect count, and virtual-thin-section count, respectively, for cementum measuring validation.

specimen	Taxon	GLG width standard deviation, transect count:											
		**2**	**3**	**4**	**5**	**6**	**7**	**8**	**9**	**10**	**11**	**12**	**13**
NHMUK PV M46234	multituberculate indet.	0.013	0.012	0.017	0.018	0.017	0.019	0.019	0.018	0.017	0.019	0.019	0.018
NHMUK PV M46503	*Phascolotherium*	0.040	0.035	0.031	0.029	0.030	0.030	0.029	0.029	0.027	0.027	0.030	0.029
NHMUK PV M46001	*Borealestes*	0.006	0.006	0.007	0.010	0.011	0.011	0.011	0.011	0.011	0.011	0.010	0.010
NHMUK PV M46570	*Simpsonodon*	0.019	0.023	0.022	0.020	0.020	0.020	0.017	0.022	0.023	0.017	0.021	0.017
NHMUK PV M46761	*Cyrtlatherium*	0.025	0.031	0.029	0.026	0.024	0.022	0.021	0.022	0.021	0.022	0.022	0.021

Supplementary Table 9. Taxa used in body mass estimation of Forest Marble taxa.

Higher order taxon	Taxon (superscript number refers to citation number - see References)	Molar count	Min. molar count	Max. molar count	m1 Length (mm)	Dentary length (mm)
Docodonta	*Agilodocodon*[114]	4	4	4	1.27	20.5
Dryolestidae	*Amblotherium*[64]	7-9	7	9	0.8	18.3
Amphidontidae	*Amphidon*[69]	4	4	4	1.3	16.9
Eutriconodonta	*Amphilestes*[115]	5	5	5	1.52	22
Amphitheriida	*Amphitherium*[68]	5-7	5	7	1.17	24.7
Zhangeotheriidae	*Anebodon*[116]	4	4	4	2.3	35
Eutriconodonta	*Aploconodon*[69]	5	5	5	1	17.9
Dryolestida	*Araeodon*[69]	5	5	5	0.9	17.4
Dryolestida	*Archaeotrigon*[69]	4	4	4	1.2	21.7
Australosphenida	*Bishops*[117]	3	3	3	1.7	18.42
Eutriconodonta	*Comodon*[69]	5	5	5	1.5	25.6
Docodonta	*Docodon*[69]	7	7	7	1.5	32.6
Eutriconodonta	*Repenomamus*[118]	4	4	4		82
Docodonta	*Docodon apoxys*[119]	7	7	7	1.58	33.2
Docodonta	*Docofossor*[120]	4	4	4	1.3	17.3
Dryolestidae	*Dryolestes*[69]	8-9	8	9	1.3	32.3
Dryolestida	*Foxraptor*[69]	5	5	5	1.2	21.7
Docodonta	*Fruitafossor*[69]	3	3	3	0.5	12.5
Eutriconodonta	*Gobiconodon borissiaki*[66]	5	5	5	2.6	35.8
Eutriconodonta	*Gobiconodon hoburensis*[66]	5	5	5	1.4	20

Docodonta	*Haldanodon*[64]	5	5	5	1.58	22.7
Amphidontidae	*Juchilestes*[121]	6	6	6	1.8	29.1
Dryolestidae	*Krebsotherium*[64]	8	8	8	1	24.3
Dryolestidae	*Laolestes*[69]	8	8	8	1.1	27.3
Dryolestida	*Paurodon*[69]	4	4	4	1.2	21.7
Eutriconodonta	*Phascolotherium*[122]	5	5	5	2	30.8
Eutriconodonta	*Priacodon*[123]	3	3	3	2.3	28.5
Multituberculata	*Psalodon*[69]	?	?	?	2.1	26.6
Metatheria	*Sinodelphys*[124]	3	3	3	1.48	20.8
Spalacotheriidae	*Spalacotherium*[64]	6	6	6	1.72	32.7
Eutriconodonta	*Spinolestes*[65]	4	4	4	1.8	30
Dryolestida	*Tathiodon*[69]	4-5	4	5	1.2	21.7
Tinodontidae	*Tinodon*[69]	4	4	4	2.2	28.6
Eutriconodonta	*Triconolestes*[69]	5	5	5	1.5	24
Eutriconodonta	*Trioracodon*[69]	3	3	3	2.6	32

Supplementary Table 10. Scaling factors between m1 length and dentary length for increasingly broad subsamples of Mesozoic fossil mammals (data presented in Supplementary Table 9), separated by number of molars within the molar row.

**Subsample**	**Scale**	***r^2***	***n***
3-4 molars	dentary length = (m1 length*10.167)+7.135	0.88	17
3-5 molars	dentary length = (m1 length*10.184)+8.026	0.84	29
3-6 molars	dentary length = (m1 length*10.187)+8.594	0.78	32
full dataset	dentary length = (m1 length*9.265)+11.214	0.65	38
4-5 molars	dentary length = (m1 length*11.573)+6.58	0.82	22
4-6 molars	dentary length = (m1 length*11.834)+6.766	0.75	25
4-9 molars	dentary length = (m1 length*10.299)+10.421	0.57	31
5-6 molars	dentary length = (m1 length*12.22)+7.004	0.74	15
5-9 molars	dentary length = (m1 length*10.448)+11.363	0.55	21
6-9 molars	dentary length = (m1 length*11.956)+12.638	0.67	9

Supplementary Table 11. Non-linear models used to characterize cementum growth.

**Model**	**formula**
Quadratic	$y = a(x)^2 – b(x) + c$
Power	$y = a(x)^b - c$
Exponential	$y = a*e^{b(x)} - c$
von Bertalanffy	$y = a*(1 – b*e^{-c(x)})$
Michaelis	$y = a(x) / (b + x)$
Logistic sigmoidal	$y = a / (1 + b*e^{(c(x)})$
Gompertz	$y = a*e^{(b * e(c(x)))}$
Gaussian	$y = a*e^{-(x-b)^2 / (2*c)^2}$
Hill's sigmoidal	$y = a / (1 + (x / b)^c)$

Data S1. Source data file containing data for charts and graphs in manuscript and supplement figures and are provided with the paper as a Source Data file.

REFERENCES AND NOTES

1. E. Newham, P. G. Gill, I. J. Corfe, New tools suggest a middle Jurassic origin for mammalian endothermy: Advances in state-of-the-art techniques uncover new insights on the evolutionary patterns of mammalian endothermy through time. *Bioessays* **44**, 2100060 (2022).

2. L. N. Weaver, H. Z. Fulghum, D. M. Grossnickle, W. H. Brightly, Z. T. Kulik, G. P. Wilson Mantilla, M. R. Whitney, Multituberculate mammals show evidence of a life history strategy similar to that of placentals, not marsupials. *Am. Nat.* **200**, 383–400 (2022).

3. R. N. O'Meara, R. J. Asher, The evolution of growth patterns in mammalian versus nonmammalian cynodonts. *Paleobiology* **42**, 439–464 (2016).

4. E. Newham, P. G. Gill, P. Brewer, M. J. Benton, V. Fernandez, N. J. Gostling, D. Haberthür, J. Jernvall, T. Kankaanpää, A. Kallonen, C. Navarro, A. Pacureanu, K. Richards, K. Robson Brown, P. Schneider, H. Suhonen, P. Tafforeau, K. A. Williams, B. Zeller-Plumhoff, I. J. Corfe, Reptile-like physiology in Early Jurassic stem-mammals. *Nat. Commun.* **11**, 5121 (2020).

5. S. Meiri, E. Levin, Revisiting life history and morphological proxies for early mammaliaform metabolic rates. *Nat. Commun.* **13**, 5562 (2022).

6. E. Newham, P. G. Gill, P. Brewer, M. J. Benton, V. Fernandez, N. J. Gostling, D. Haberthür, J. Jernvall, T. Kankaanpää, A. Kallonen, C. Navarro, A. Pacureanu, K. Richards, K. Robson Brown, P. Schneider, H. Suhonen, P. Tafforeau, K. A. Williams, B. Zeller-Plumhoff, I. J. Corfe, Reply to: Revisiting life history and morphological proxies for early mammaliaform metabolic rates. *Nat. Commun.* **13**, 5564 (2022).

7. J. Avaria-Llautureo, C. E. Hernández, E. Rodríguez-Serrano, C. Venditti, The decoupled nature of basal metabolic rate and body temperature in endotherm evolution. *Nature* **572**, 651–654 (2019).

8. R. Araújo, R. David, J. Benoit, J. K. Lungmus, A. Stoessel, P. M. Barrett, J. A. Maisano, E. Ekdale, M. Orliac, Z. X. Luo, A. Martinelli, E. A. Hoffman, C. A. Sidor, R. M. S. Martins, F.

Spoor, K. D. Angielczyk, Inner ear biomechanics reveals a Late Triassic origin for mammalian endothermy. *Nature* **607**, 726–731 (2022).

9. A. J. Hulbert, R. Pamplona, R. Buffenstein, W. A. Buttemer, Life and death: Metabolic rate, membrane composition, and life span of animals. *Physiol. Rev.* **87**, 1175–1213 (2007).

10. C. R. White, N. F. Phillips, R. S. Seymour, The scaling and temperature dependence of vertebrate metabolism. *Biol. Lett.* **2**, 125–127 (2005).

11. E. A. Hoffman, T. B. Rowe, Jurassic stem-mammal perinates and the origin of mammalian reproduction and growth. *Nature* **561**, 104–108 (2018).

12. H. S. Mumby, S. N. Chapman, J. A. H. Crawley, K. U. Mar, W. Htut, A. Thura Soe, H. H. Aung, V. Lummaa, Distinguishing between determinate and indeterminate growth in a long-lived mammal. *BMC Evol. Biol.* **15**, 214 (2015).

13. S. Nicol, N. A. Andersen, The life history of an egg-laying mammal, the echidna (*Tachyglossus aculeatus*). *Ecoscience* **14**, 275–285 (2007).

14. M. Köhler, S. Moyà-Solà, Physiological and life history strategies of a fossil large mammal in a resource-limited environment. *Proc. Natl. Acad. Sci. U.S.A.* **106**, 20354–20358 (2009).

15. S. Herculano-Houzel, Longevity and sexual maturity vary across species with number of cortical neurons, and humans are no exception. *J. Comp. Neurol.* **527**, 1689–1705 (2019).

16. E. M. Zullinger, R. E. Ricklefs, K. H. Redford, G. M. Mace, Fitting sigmoidal equations to mammalian growth curves. *J. Mammal.* **65**, 607–636 (1984).

17. S. Ray, J. Botha, J. A. Chinsamy, Bone histology and growth patterns of some nonmammalian therapsids. *J. Vertebr. Paleontol.* **24**, 634–648 (2004).

18. A. Chinsamy, J. H. Hurum, Bone microstructure and growth patterns of early mammals. *Acta Palaeontol. Pol.* **51**, 325–338 (2006).

19. R. N. O’Meara, W. Dirks, A. G. Martinelli, Enamel formation and growth in non-mammalian cynodonts. *R. Soc. Open Sci.* **5**, 172293 (2018).

20. J. H. Hurum, A. Chinsamy-Turan, “The radiation, bone histology, and biology of early mammals” in *Forerunners of Mammals: Radiation, Histology, Biology*, A. Chinsamy-Turan, Ed. (Indiana Univ. Press, 2011).

21. Z. X. Luo, Z. Kielan-Jaworowska, R. L. Cifelli, Evolution of dental replacement in mammals. *Bull. Carnegie Mus. Nat. Hist.* **2004**, 159–175 (2004).

22. G. Klevezal, *Recording Structures of Mammals* (CRC Press, 1995).

23. S. Naji, T. Colard, J. Blondiaux, B. Bertrand, E. d’Incau, J. P. Bocquet-Appel, Cementochronology, to cut or not to cut? *Int. J. Paleopathol.* **15**, 113–119 (2016).

24. R. A. Close, M. Friedman, G. T. Lloyd, R. B. Benson, Evidence for a mid-Jurassic adaptive radiation in mammals. *Curr. Biol.* **25**, 2137–2142 (2015).

25. A. W. Crompton, T. Owerkowicz, B. A. Bhullar, C. Musinsky, Structure of the nasal region of non-mammalian cynodonts and mammaliaforms: Speculations on the evolution of mammalian endothermy. *J. Vertebr. Paleontol.* **37**, e1269116 (2017).

26. D. E. Lieberman, Life history variables preserved in dental cementum microstructure. *Science* **261**, 1162–1164 (1993).

27. E. Newham, I. J. Corfe, K. R. Brown, N. J. Gostling, P. G. Gill, P. Schneider, Synchrotron radiation-based X-ray tomography reveals life history in primate cementum incrementation. *J. R. Soc. Interface* **17**, 20200538 (2020).

28. G. A. Klevezal, B. S. Stewart, Patterns and calibration of layering in tooth cementum of female northern elephant seals, *Mirounga angustirostris*. *J. Mamm.* **75**, 483–487 (1994).

29. V. R. Von Biela, J. W. Testa, V. A. Gill, J. M. Burns, Evaluating cementum to determine past reproduction in northern sea otters. *J. Wildl. Manage* **72**, 618–624 (2008).

30. S. Medill, A. E. Derocher, I. Stirling, N. Lunn, Reconstructing the reproductive history of female polar bears using cementum patterns of premolar teeth. *Polar Biol.* **33**, 115–124 (2010).

31. T. A. Wittmann, C. Izzo, Z. A. Doubleday, J. McKenzie, S. Delean, B. M. Gillanders, Reconstructing climate-growth relations from the teeth of a marine mammal. *Mar. Biol.* **163**, 1–11 (2016).

32. E. Newham, S. Naji, "Identifying life-history events in dental cementum: A literature review" in *Dental Cementum in Anthropology*, S. Naji, W. Rendu, L. Gourichon, Eds. (Cambridge Univ. Press, 2022).

33. D. I. Whiteside, C. J. Duffin, P. G. Gill, J. E. A. Marshall, M. J. Benton, The Late Triassic and Early Jurassic fissure faunas from Bristol and South Wales: Stratigraphy and setting. *Acta Pal. Pol.* **67**, 257–287 (2016).

34. E. F. Freeman, Kirtlington Quarry: Its history and geology and the search for early mammals. *Mercian Geol.* **19**, 232 (2019).

35. T. Martin, Mammalian fauna of the Late Jurassic Guimarota ecosystem. *PE-APA* **7**, 123–126 (2001).

36. E. Newham, P. G. Gill, K. Robson Brown, N. J. Gostling, I. J. Corfe, P. Schneider, A robust, semi-automated approach for counting cementum increments imaged with synchrotron X-ray computed tomography. *PLOS ONE* **16**, e0249743 (2021).

37. D. C. Jhwueng, C. P. Wang, Phylogenetic curved optimal regression for adaptive trait evolution. *Entropy* **23**, 218 (2021).

38. I. Scharf, A. Feldman, M. Novosolov, M. Novosolov, D. Pincheira-Donoso, I. Das, M. Böhm, P. Uetz, O. Torres-Carvajal, A. Bauer, U. Roll, S. Meiri, Late bloomers and baby boomers: Ecological drivers of lifespan in squamates and the tuatara. *Glob. Ecol. Biogeogr.* **24**, 396–405 (2015).

39. B. G. Lovegrove, A phenology of the evolution of endothermy in birds and mammals. *Biol. Rev. Camb. Philos. Soc.* **92**, 1213–1240 (2017).

40. A. K. Huttenlocker, J. Botha-Brink, Bone microstructure and the evolution of growth patterns in Permo-Triassic therocephalians (Amniota, Therapsida) of South Africa. *PeerJ* **2**, e325 (2014).

41. J. Botha-Brink, D. Codron, A. K. Huttenlocker, K. Angielczyk, M. Ruta, Breeding young as a survival strategy during Earth's greatest mass extinction. *Sci. Rep.* **6**, 24053 (2016).

42. T. B. Rowe, T. E. Macrini, Z. X. Luo, Fossil evidence on origin of the mammalian brain. *Science* **332**, 955–957 (2011).

43. P. Frýdlová, J. Mrzílková, M. Šeremeta, J. Křemen, J. Dudák, J. Žemlička, B. Minnich, K. Kverková, P. Němec, P. Zach, D. Frynta, Determinate growth is predominant and likely ancestral in squamate reptiles. *Proc. Royal Soc. B* **287**, 20202737 (2020).

44. M. Köhler, N. Marín-Moratalla, X. Jordana, R. Aanes, Seasonal bone growth and physiology in endotherms shed light on dinosaur physiology. *Nature* **487**, 358–361 (2012).

45. A. D. Yoder, C. R. Campbell, M. B. Blanco, M. Dos Reis, J. U. Ganzhorn, S. M. Goodman, K. E. Hunnicutt, P. A. Larson, P. M. Kappeler, R. M. Rasoloarison, J. M. Ralison, D. L. Swofford, D. W. Weisrock, Geogenetic patterns in mouse lemurs (genus *Microcebus*) reveal the ghosts of Madagascar's forests past. *Proc. Natl Acad. Sci. U.S.A.* **113**, 8049–8056 (2016).

46. G. F. Funston, P. E. dePolo, J. T. Sliwinski, M. Dumont, S. L. Shelley, L. E. Pichevin, N. J. Cayzer, J. R. Wible, T. E. Williamson, J. W. B. Rae, S. L. Brusatte, The origin of placental mammal life histories. *Nature* **610**, 107–111 (2022).

47. G. P. Wilson, A. R. Evans, I. J. Corfe, P. D. Smits, M. Fortelius, J. Jernvall, Adaptive radiation of multituberculate mammals before the extinction of dinosaurs. *Nature* **483**, 457–460 (2012).

48. A. K. Huttenlocker, Body size reductions in nonmammalian eutheriodont therapsids (Synapsida) during the end-Permian mass extinction. *PLOS ONE* **9**, e87553 (2014).

49. P. O. Montiglio, M. Dammhahn, G. Dubuc Messier, D. Réale, The pace-of-life syndrome revisited: The role of ecological conditions and natural history on the slow-fast continuum. *Behav. Ecol. Sociobiol.* **72**, 116 (2018).

50. D. Réale, B. Y. Gallant, M. Leblanc, M. Festa-Bianchet, Consistency of temperament in bighorn ewes and correlates with behaviour and life history. *Anim. Behav.* **60**, 589–597 (2000).

51. P. G. Gill, M. A. Purnell, N. Crumpton, K. Robson Brown, N. Gostling, M. Stampanoni, E. Rayfield. Dietary specializations and diversity in feeding ecology of the earliest stem mammals. *Nature* **512** 303–305 (2014).

52. S. Wills, E. Bernard, P. Brewer, C. Underwood, D. Ward, Palaeontology, stratigraphy and sedimentology of Woodeaton Quarry (Oxfordshire) and a new microvertebrate site from the White Limestone Formation (Bathonian, Jurassic). *Proc. Geol. Assoc.* **130**, 170–186 (2019).

53. E. F. Freeman, A Middle Jurassic mammal bed from Oxfordshire. *Palaeontology* **22**, 135–166 (1979).

54. T. Martin, B. Krebs, Eds., *Guimarota: A Jurassic Ecosystem* (Verlag Dr. Friedrich Pfeil, 2000).

55. D. Paganin, S. C. Mayo, T. E. Gureyev, P. R. Miller, S. W. Wilkins, Simultaneous phase and amplitude extraction from a single defocused image of a homogeneous object. *J. Microsc.* **206**, 33–40 (2002).

56. F. Marone, A. Studer, H. Billich, L. Sala, M. Stampanoni, Towards on-the-fly data post-processing for real-time tomographic imaging at TOMCAT. *Adv. Struct. Chem. Imaging* **3**, 1 (2017).

57. F. Mirone, E. Brun, E. Gouillart, P. Tafforeau, J. Kieffer, The PyHST2 hybrid distributed code for high speed tomographic reconstruction with iterative reconstruction and a priori knowledge capabilities. *Nucl. Instrum. Methods Phys. Res. B* **324**, 41–48 (2014).

58. C. A. Schneider, W. S. Rasband, K. W. Eliceiri, NIH Image to ImageJ: 25 years of image analysis. *Nat. Methods* **9**, 671–675 (2001).

59. E. Newham, “Exploring the Use of X-ray Tomography for the Quantification of Cementum Growth Patterns Across the Mammal Phylogeny,” thesis, University of Southampton (2018).

60. A. Le Cabec, N. K. Tang, V. Ruano Rubio, S. Hillson, Nondestructive adult age estimation: Visualizing cementum annulations in a known age historical human assemblage using synchrotron X-ray microtomography. *Am. J. Phys. Anthropol.* **168**, 25–44 (2019).

61. G. Mani-Caplazi, G. Schulz, H. Deyhle, G. Hotz, W. Vach, U. Wittwer-Backofen, B. Müller, Imaging of the human tooth cementum ultrastructure of archaeological teeth, using hard X-ray microtomography to determine age-at-death and stress periods, in *Proceedings of SPIE Vol. 10391*, (SPIE, 2017), 8 pp.

62. Z. Kielan-Jaworowska, R. L. Cifelli, Z. X. Luo, *Mammals from the Age of Dinosaurs: Origins, Evolution, and Structure* (Columbia Univ. Press, 2004).

63. F. A. Jenkins Jr., C. R. Schaff, The Early Cretaceous mammal *Gobiconodon* (Mammalia, Triconodonta) from the Cloverly Formation in Montana. *J. Vertebr. Paleontol.* **8**, 1–24 (1988).

64. A. Lopatin, A. Averianov, *Gobiconodon* (Mammalia) from the Early Cretaceous of Mongolia and revision of Gobiconodontidae. *J. Mamm. Evol* **22**, 17–43 (2015).

65. T. Martin, J. Marugán-Lobón, R. Vullo, H. Martín-Abad, Z. X. Luo, A. D. Buscalioni, A Cretaceous eutriconodont and integument evolution in early mammals. *Nature* **526**, 380–384 (2015).

66. K. R. K. Jäger, R. L. Cifelli, T. Martin, Tooth eruption in the Early Cretaceous British mammal *Triconodon* and description of a new species. *Pap. Palaeontol.* **7**, 1065–1080 (2021).

67. M. Purnell, O. Seehausen, F. Galis, Quantitative three-dimensional microtextural analyses of tooth wear as a tool for dietary discrimination in fishes. *J. R. Soc. Interface* **9**, 2225–2233 (2012).

68. E. Panciroli, R. Benson, R. Butler, New partial dentaries of amphitheriid mammalian *Palaeoxonodon ooliticus* from Scotland, and posterior dentary morphology in early cladotherians. *Acta Pal. Pol.* **63**, 197–206 (2018).

69. J. R. Foster, Preliminary body mass estimates for mammalian genera of the Morrison Formation (Upper Jurassic, North America). *PaleoBios* **28**, 114–122 (2009).

70. E. C. Kirk, S. Hoffmann, A. D. Kemp, D. W. Krause, P. M. O'Connor, Sensory anatomy and sensory ecology of *Vintana sertichi* (Mammalia, Gondwanatheria) from the Late Cretaceous of Madagascar. *J. Vertebr. Paleontol.* **34**, 203–222 (2014).

71. E. Panciroli, R. B. Benson, Z. X. Luo, The mandible and dentition of *Borealestes serendipitus* (Docodonta) from the Middle Jurassic of Skye, Scotland. *J. Vertebr. Paleontol.* **39**, e1621884 (2019).

72. Ø. Hammer, D. A. Harper, P. D. Ryan, PAST: Paleontological statistics software package for education and data analysis. *Palaeontol. Electron.* **4**, 9 (2001).

73. J. B. Smaers, F. J. Rohlf, Testing species' deviation from allometric predictions using the phylogenetic regression. *Evolution* **70**, 1145–1149 (2016).

74. C. A. Spinage, Incremental cementum lines in the teeth of tropical African mammals. *J. Zool.* **178**, 117–131 (1976).

75. T. S. Myers, N. J. Tabor, L. L. Jacobs, O. Mateus, Palaeoclimate of the Late Jurassic of Portugal: Comparison with the western United States. *Sedimentology* **59**, 1695–1717 (2012).

76. P. G. Gill, L. K. Säilä, I. J. Corfe, T. J. Challans, M. Williams, W. A. Clemens, The fauna and palaeoenvironment of St. Bride's Island fissure fills of South Wales, in *Ninth International Symposium on Mesozoic Terrestrial Ecosystems and Biota, Abstracts and Proceedings,* P. M. Barrett, S. E. Evans, Eds. (Natural History Museum, 2006), pp. 48–51.

77. R. E. Ricklefs, M. Wikelski, The physiology/life-history nexus. *Trends Ecol. Evol.* **17**, 462–468 (2002).

78. M. Wolf, G. Sander van Doorn O. Leimar, F. J. Weissing, Life-history trade-offs favour the evolution of animal personalities. *Nature* **447**, 581–584 (2007).

79. J. A. Stamps, Growth-mortality tradeoffs and "personality traits" in animals. *Ecol. Lett.* **10**, 355–363 (2007).

80. V. Careau, O. R. P. Bininda, D. W. Thomas, D. Réale, M. M. Humphries, Exploration strategies map along fast-slow metabolic and life-history continua in muroid rodents. *Funct. Ecol.* **23**, 150–15 (2009).

81. V. Careau, D. Thomas, F. Pelletier, *L. Turki*, F. Landry, D. Garant, D. Réale, Genetic correlation between resting metabolic rate and exploratory behaviour in deer mice (*Peromyscus maniculatus*). *J. Evol. Biol.* **24**, 2153–2163 (2011).

82. M. E. Timonin, C. J. Carrière, A. D. Dudych, J. G. W. Latimer, S. T. Unruh, C. K. R. Willis, Individual differences in the behavioural responses of meadow voles to an unfamiliar environment are not correlated with variation in resting metabolic rate. *J. Zool.* **284**, 198–205 (2011).

83. D. Réale, M. Festa-Bianchet, Predator-induced natural selection on temperament in bighorn ewes. *Anim. Behav.* **65**, 463–470 (2003).

84. D. Réale, J. Martin, D. W. Coltman, J. Poissant, M. Festa-Bianchet, Male personality, life-history strategies and reproductive success in a promiscuous mammal. *J. Evol. Biol.* **22**, 1599–1607 (2009).

85. P. Bergeron, P.-O.Montiglio, D. Réale, M. M. Humphries, O. Gimenez, D. Garant, Disruptive viability selection on adult exploratory behaviour in eastern chipmunks. *J. Evol. Biol.* **26**, 766–774 (2013).

86. P. T. Niemelä, N. J. Dingemanse, N. Alioravainen, A. Vainikka, R. Kortet, Personality pace-of-life hypothesis: Testing genetic associations among personality and life history. *Behav. Ecol.* **24**, 935–941 (2013).

87. T. Müller, C. Müller, Behavioural phenotypes over the lifetime of a holometabolous insect. *Front. Zool.* **12**, S8 (2015).

88. P. A. Morris, A method for determining absolute age in the hedgehog. *J. Zool.* **161**, 277–281 (1970).

89. B. Frylestam, T von Schantz, Age determination of European hares based on periosteal growth lines. *Mamm. Rev.* **7**, 151–154 (1977).

90. J. Castanet, S. Croci, F. Aujard, M. Perret, J. Cubo, E. de Margerie, Line of arrested growth in bone and age estimation in a small primate: *Microcebus murinus*. *J. Zool. Lond.* **263**, 31–39 (2004).

91. F. Dyason, Dental cementum analysis of macropod teeth in Australia: A methodological enquiry. *Quat. Aust.* **25**, 12 (2008).

92. Y. M. Lam, "The applicability of cementum increment analysis to seasonality studies on the South African Cape", thesis, State University of New York at Stony Brook (2002).

93. V. Veiberg, E. B. Nilsen, C. M. Rolandsen, M. Heim, R. Andersen, F. Holmstrøm, E. L. Meisingset, E. J. Solberg, The accuracy and precision of age determination by dental cementum annuli in four northern cervids. *Eur. J. Wildl. Res.* **66**, 1–11 (2020).

94. C. M. Wall-Scheffler, R. A. Foley, Digital cementum luminance analysis (DCLA): A tool for the analysis of climatic and seasonal signals in dental cementum. *Int. J. Osteoarchaeol.* **18**, 11–27 (2008).

95. H. Grue, B. Jensen, Annual cementum structures in canine teeth in arctic foxes (*Alopex lagopus* L.) from Greenland and Denmark. *Dan. Rev. Game Biol.* **10**, 1–12 1976).

96. M. P. English, P. Morris, *Trichophyton mentagrophytes var. erinacei* in hedgehog nests. *Sabouraudia* **7**, 118–121 (1969).

97. A. Hakeem, R. Sandoval, M. Jones, J. Allman, "Brain and life span in primates" in *Handbook of the Psychology of Aging*, J. Birren, Ed. (Academic Press, 1996), pp. 78–104.

98. I. Stirling, W. Calvert, D. Andriashek, Population ecology studies of the polar bear in the area of southeastern Baffin Island. *Occas. Pap. Can. Wild. Serv.* **44**, 33 (1980).

99. D. Hunsaker, D. Shupe, "Behavior of new world marsupials" in *The Biology of Marsupials* (Academic Press, 1977), pp. 279–347.

100. B. Grzimek, N. Schlager, D. Olendorf, *Grzimek's Animal Life Encyclopedia* (Gale, 2003).

101. R. L. Peterson, A review of the general life history of the moose. *Nat. Can.* **101**, 9–21 (1974).

102. D. W. Müller, J. M. Gaillard, L. B. Lackey, J. M. Hatt, M. Clauss, Comparing life expectancy of three deer species between captive and wild populations. *Eur. J. Wildl. Res.* **56**, 205–208 (2010).

103. L. D. Mech, Wolf longevity in the wild. *Endanger. Species Bull.* **14**, 8 (1989).

104. R. Weigl, *Longevity of Mammals in Captivity: From the Living Collections of the World* (E. Schweizerbart'sche, 2005).

105. R. M. Nowak, *Walker's Mammals of the World* (Johns Hopkins Univ. Press, 1991).

106. C. J. Martinka, K. L. McArthur, *Bears, Their Biology and Management: A Selection of Papers from the Fourth International Conference on Bear Research and Management Held at Kalispell, Montana, USA, February 1977* (Bear Biology Association, 1980).

107. E. Yensen, P. Sherman, “Ground squirrels *Spermophilus* and *Ammospermophilus* species” in *Wild Mammals of North America: Biology, Management, and Conservation* (Johns Hopkins Univ. Press, ed. 2, 2003).

108. J. L. Mulder, Longevity records in the red fox. *Lutra* **47**, 51–52 (2004).

109. A. Myllymäki, Demographic mechanisms in the fluctuating populations of the field vole *Microtus agrestis*. *Oikos* **29**, 468–493 (1977).

110. G. A. Sacher, R. W. Hart, Longevity, aging, and comparative cellular and molecular biology of the house mouse, *Mus musculus*, and the white-footed mouse, *Peromyscus leucopus*. *Birth Defects Orig. Artic. Ser.* **14**, 71–96 (1978).

111. L. M. Rotterman, T. Simon-Jackson, “Sea otter (*Enhydra lutris*)” in *Selected Marine Mammals of Alaska: Species Accounts with Research and Management Recommendations* (Marine Mammal Commission, 1988).

112. V. Geist, *Mountain Sheep: A Study in Behavior and Evolution* (University of Chicago Press, 1971).

113. A. M. Audet, C. B. Robbins, S. Larivière, *Alopex lagopus*. *Mamm. Spec.* **713**, 1–10 (2002).

114. Q. J. Meng, Q. Ji, Y. G. Zhang, D. Liu, D. M. Grossnickle, Z. X. Luo, An arboreal docodont from the Jurassic and mammaliaform ecological diversification. *Science* **347**, 764–768 (2015).

115. T. Martin, “Mesozoic mammals—Early mammalian diversity and ecomorphological adaptations” in *Handbook of Zoology: Mammalian Evolution, Diversity and Systematics*, F. E. Zachos, R. J. Asher, Eds. (De Gruyter, 2018).

116. S. Bi, X. Zheng, J. Meng, X. Wang, N. Robinson, B. Davis, A new symmetrodont mammal (Trechnotheria: Zhangheotheriidae) from the Early Cretaceous of China and trechnotherian character evolution. *Sci. Rep.* **6**, 1–9 (2016).

117. T. H. Rich, P. Vickers-Rich, T. F. Flannery, D. Pickering, L. Kool, A. M. Tait, E. M. G. Fitzgerald, A fourth Australian Mesozoic mammal locality. *Mus. North. Ariz.* **65**, 677–681 (2009).

118. Y. Hu, J. Meng, Y. Wang, C. Li, Large Mesozoic mammals fed on young dinosaurs. *Nature* **433**, 149–152 (2005).

119. G. W. Rougier, A. S. Sheth, K. Carpenter, L. Appella-Guiscafre, B. M. Davis, A new species of *Docodon* (Mammaliaformes: Docodonta) from the Upper Jurassic Morrison Formation and a reassessment of selected craniodental characters in basal mammaliaforms. *J. Mamm. Evol.* **22**, 1–16 (2015).

120. Z. X. Luo, Q. J. Meng, Q. Ji, D. Liu, Y. G. Zhang, A. I. Neander, Evolutionary development in basal mammaliaforms as revealed by a docodontan. *Science* **347**, 760–764 (2015).

121. C. L. Gao, G. P. Wilson, Z. X. Luo, A. M. Maga, Q. Meng, X. Wang, A new mammal skull from the Lower Cretaceous of China with implications for the evolution of obtuse-angled molars and 'amphilestid' eutriconodonts. *Proc. Biol. Sci.* **277**, 237–246 (2010).

122. P. F. Wilson, J. Stott, J. M. Warnett, A. Attridge, M. P. Smith, M. Williams, Evaluation of touchable 3D-printed replicas in museums. *Curator* **60**, 445–465 (2017).

123. K. R. K. Jäger, R. L. Cifelli, T. Martin, Molar occlusion and jaw roll in early crown mammals. *Sci. Rep.* **10**, 22378 (2020).

124. S. Bi, X. Zheng, X. Wang, N. E. Cignetti, S. Yang, J. R. Wible, An Early Cretaceous eutherian and the placental-marsupial dichotomy. *Nature* **558**, 390–395 (2018).
